# Molecular architecture and domain arrangement of the placental malaria protein VAR2CSA suggests a model for carbohydrate binding

**DOI:** 10.1074/jbc.RA120.014676

**Published:** 2021-01-13

**Authors:** Maria C. Bewley, Lovely Gautam, Mashanipalya G. Jagadeeshaprasad, D. Channe Gowda, John M. Flanagan

**Affiliations:** Department of Biochemistry and Molecular Biology, The Pennsylvania State University College of Medicine, Milton S. Hershey Medical Center, Hershey, Pennsylvania, USA

**Keywords:** placental malaria, *Plasmodium falciparum* erythrocyte protein 1, VAR2CSA, chondroitin 4-sulfate, small angle x-ray scattering, electron microscopy, malaria, carbohydrate function, electron microscopy (EM), protein structure, mass spectrometry, *Plasmodium falciparum* erythrocyte protein-1

## Abstract

VAR2CSA is the placental-malaria–specific member of the antigenically variant *Plasmodium falciparum* erythrocyte membrane protein 1 (PfEMP1) family. It is expressed on the surface of *Plasmodium falciparum-*infected host red blood cells and binds to specific chondroitin-4-sulfate chains of the placental proteoglycan receptor. The functional ∼310 kDa ectodomain of VAR2CSA is a multidomain protein that requires a minimum 12-mer chondroitin-4-sulfate molecule for specific, high affinity receptor binding. However, it is not known how the individual domains are organized and interact to create the receptor-binding surface, limiting efforts to exploit its potential as an effective vaccine or drug target. Using small angle X-ray scattering and single particle reconstruction from negative-stained electron micrographs of the ectodomain and multidomain constructs, we have determined the structural architecture of VAR2CSA. The relative locations of the domains creates two distinct pores that can each accommodate the 12-mer of chondroitin-4-sulfate, suggesting a model for receptor binding. This model has important implications for understanding cytoadherence of infected red blood cells and potentially provides a starting point for developing novel strategies to prevent and/or treat placental malaria.

Several species of *Plasmodium* parasites cause malaria in humans; however, the deadliest forms of malaria, including cerebral and placental malaria, are caused by *Plasmodium falciparum* (Pf) (1, 2, 3). The severity of Pf malaria is a consequence of the sequestration of parasite-infected red blood cells (IRBCs) in the vital organs of the host, leading to inflammation and fatal pathological conditions. Parasite sequestration is mediated by a family of ∼60 antigenically variant proteins, called Pf erythrocyte membrane protein 1 (PfEMP1), expressed on the IRBC surface, which are capable of binding to several host cell adhesion molecules in the vasculature of various organs (4, 5, 6, 7, 8, 9, 10, 11, 12, 13, 14, 15). Multiplication of parasites sequestered in host organs leads to microcirculatory obstruction, hypoxia, inflammation, organ dysfunction, and the severe pathologies of Pf malaria (16, 17, 18, 19, 20, 21).

As a result of frequent Pf infections, most adults in malaria endemic areas have acquired protective anti-PfEMP1 antibodies to parasites strains expressing various PfEMP1s, except the placental-specific one, VAR2CSA (22). During the first pregnancy, Pf exploits the development of the placenta to overcome their pre-existing immunity by expressing VAR2CSA and sequestering in the placenta by specifically binding to the chondroitin-4-sulfate (CSA) chains of the chondroitin sulfate proteoglycan (CSPG) receptors. Subsequently, rapid parasite multiplication contributes to placental dysfunction, maternal anemia, preterm delivery, low neonate weight, and maternal and pediatric morbidity and mortality (23, 24); collectively these conditions are referred to as placental malaria (PM) (25, 26, 27, 28). Women infected with Pf during prior pregnancies produce anti-VAR2CSA antibodies (29) and thus resist PM development in subsequent pregnancies (30), suggesting that VAR2CSA is a suitable therapeutic target. Even though the parasite expresses polymorphic VAR2CSA, which poses challenges in the development of long-lasting efficacious vaccines against diverse CSA-binding isolates, placental sequestration of IRBCs in the intervillous spaces of the placenta and syncytiotrophoblast surface is an obligate step in the pathology of PM (31, 32, 33, 34, 35) that is facilitated by VAR2CSA binding to the host CSA chains of the CSPGs. Therefore, understanding the nature of the CSA-VAR2CSA interaction is a critical step toward developing effective therapeutic strategies that prevent IRBC cytoadherence in the placenta (36).

VAR2CSA is a ∼350-kDa membrane protein consisting of a large ∼310-kDa nonglycosylated extracellular ectodomain, a single pass transmembrane helix, and a ∼42-kDa cytoplasmic acidic terminal sequence that interacts with a number of host cell proteins (34). The functional CSA-binding ectodomain is comprised of six Duffy binding-like (DBL) domains (DBL1x, DBL2x, DBL3x, DBL4ε, DBL5ε, and DBL6ε) and two interdomains (ID1 and ID2/CIDR_PAM_), connected to each other by short linker sequences (Fig. 1*A*). Together, these domains form a high affinity ligand-binding site with specificity for a minimum 12-mer CSA that has a characteristic, low C4 sulfate content (lsCSA) (31, 33, 37). To gain structural information, small angle X-ray scattering (SAXS) studies were performed using human embryonic kidney (HEK) cell-expressed, engineered, non-*N*-glycosylated and baculovirus-expressed glycosylated ectodomains (38, 39). However, the resultant molecular envelopes were relatively featureless, preventing identification of individual domains or insight into carbohydrate binding. To date, there is no high-resolution structure of the VAR2CSA ectodomain, although crystal structures of *Escherichia coli*-expressed DBL3x (PDB codes 3BQI and 3CML) (40, 41), DBL6ε (2WAU and 2Y8D), (42, 43), and the tandem DBL3x-DBL4ε domains (4P1T) (44) reveal a conserved fold comprised of three subdomains that are stabilized by multiple intradomain disulfide bonds. However, the detailed tertiary structure that forms the functional, high affinity CSA-binding surface remains unclear. It is generally agreed that domains DBL1x, ID1, DBL2x, and the N-terminal half of ID2 (ID2a) are involved (38, 39, 45, 46, 47); but it is unclear whether they comprise the entire surface area for CSA binding. Therefore, to understand the details of carbohydrate binding it is essential to know the structural architecture of VAR2CSA.

**Figure 1:**
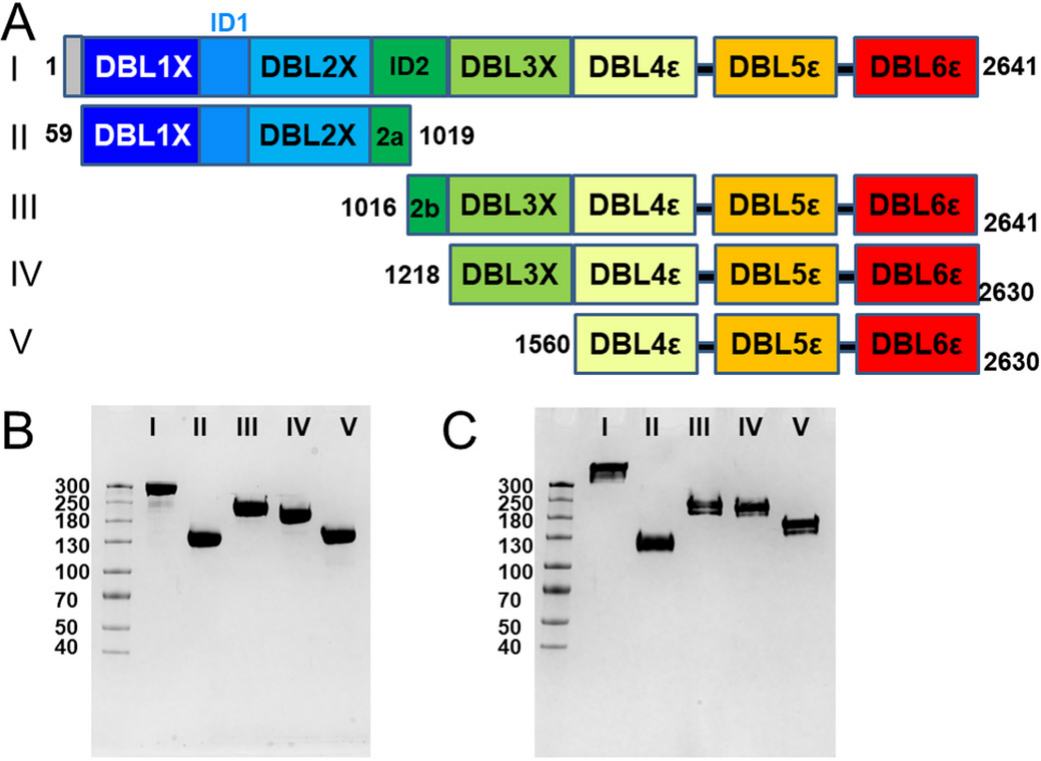
**VAR2CSA and its deletion constructs expressed in HEK 293F cells and SDS-PAGE of the purified recombinant VAR2CSA proteins.***A,* schematic indicating the locations of DBL domains for constructs I–V. For each construct, the numbers at either end denote the beginning and ending amino acid sequence numbers. The noncleavable C-terminal cMyc tag and His_6_ tag, as coded in the commercial vector, was replaced with a TEV-cleavable 3× FLAG tag and a His_6_ tag, as defined in Table S2 GBLOCK2, for constructs III and V. In addition, P1 contains a glycine and threonine residue inserted between native residues 58 and 59 as a cloning artifact. *B,* purified proteins (each 2 µg/lane), electrophoresed using 4–20% gradient gels (1 mm thick) under reducing conditions, and *C,* nonreducing conditions are shown. In each gel, the lanes are: *M*, molecular weight markers; *I*, NTS-DBL6ε (∼310 kDa); *II*, DBL1x-ID2a (∼125 kDa); *III*, ID2b-DBL6ε (∼200 kDa); *IV*, DBL3x-DBL6ε (∼180 kDa); *V*, DBL4ε-DBL6ε (∼130 kDa). The molecular mass marker (kDa) standards are indicated in the *left* margin.

In the present study, we have expressed and characterized, structurally and functionally, the VAR2CSA ectodomain and a set of N- and C-terminal deletion constructs (Fig. 1). These mammalian-expressed, recombinant proteins are folded and thermally stable. NTS-DBL6ε and DBL1x-ID2a specifically bind the lsCSA chains of CSPG with nanomolar affinity and ID2b-DBL6ε, DBL3x-DBL6ε, and DBL4ε-DBL6ε do not show measurable binding. Using a combination of size-exclusion chromatography in-line with SAXS (SEC-SAXS), single particle reconstruction of negative-stained electron micrographs and basic homology modeling, we have determined the relative locations of the DBL domains and produced a validated model of the VAR2CSA ectodomain. Importantly, these studies reveal, for the first time, a defined molecular shape with distinctive pores that transverse the molecule and suggest a credible model for CSA binding.

## Results

### Production, purification, and characterization of VAR2CSA constructs

The codon-optimized synthetic gene encoding the VAR2CSA ectodomain (NTS-DBL6ε) of Pf 3D7 strain and four deletion constructs corresponding to DBL1x-ID2a, ID2b-DBL6ε, DBL3x-DBL6ε, and DBL4ε-DBL6ε (as defined in Fig. 1*A* and Tables S1 and S2) were expressed in HEK Freestyle 293-F (HEK 293F) cells. The proteins are produced as secreted monomers and purified using a combination of ultrafiltration, nickel affinity, cation exchange, and size-exclusion chromatography. After purification and SDS-PAGE under reducing conditions, a single band at the expected apparent molecular mass was observed for each protein (Fig. 1*B*). Similarly, under nonreducing conditions a single band was observed for each protein (Fig. 1*C*). The purity of each protein construct, as assessed by the SDS-PAGE band profiles, is ∼98%. Yields of the purified recombinant proteins range from 0.5 to 0.7 mg/liter for NTS-DBL6ε to 14 mg/liter for DBL4ε-DBL6ε. Several additional deletion constructs (DBL1x-DBL5ε, DBL1-ID2b, ID1-DBL6ε, and ID1-ID2) were tested for protein production in our system, but these either failed to express or to purify as folded monomers and thus were not analyzed further.

To assess the folding of the purified recombinant proteins, far UV CD spectra were recorded at 25 °C and evaluated. A strong maximum at 200 nm and troughs at 208 and 222 nm were observed (Fig. S1A) that were characteristic of proteins containing substantial α-helical secondary structures. The spectra are similar to results reported for VAR2CSA constructs DBL6ε, DBL4ε-DBL6ε, and DBL1x-DBL6ε (38). The stability of each construct was investigated by following the changes in ellipticity at 222 nm as a function of temperature between 25 and 85 °C. In all cases, a transition occurs between 70 and 75 °C (Fig. S1B), demonstrating that these constructs are thermally stable. The observed transition temperatures are similar to those reported earlier for analogous constructs (38). However, for each construct, the spectrum collected at 90 °C shows residual ellipticity at 208 and 222 nm (Fig. S1, C–G), demonstrating that unfolding is incomplete and preventing calculation of a thermodynamically-justified value for *T_m_*. Indeed, the complete unfolding of NTS-DBL6ε is achieved only in the presence of the reducing agent tris(2-carboxyethyl) phosphine (Fig. S1H). These results are consistent with the constructs being stable, as expected for domains containing a large number of disulfide bonds (48).

### SAXS analysis suggests that the VAR2CSA ectodomain is compact

**Figure 2:**
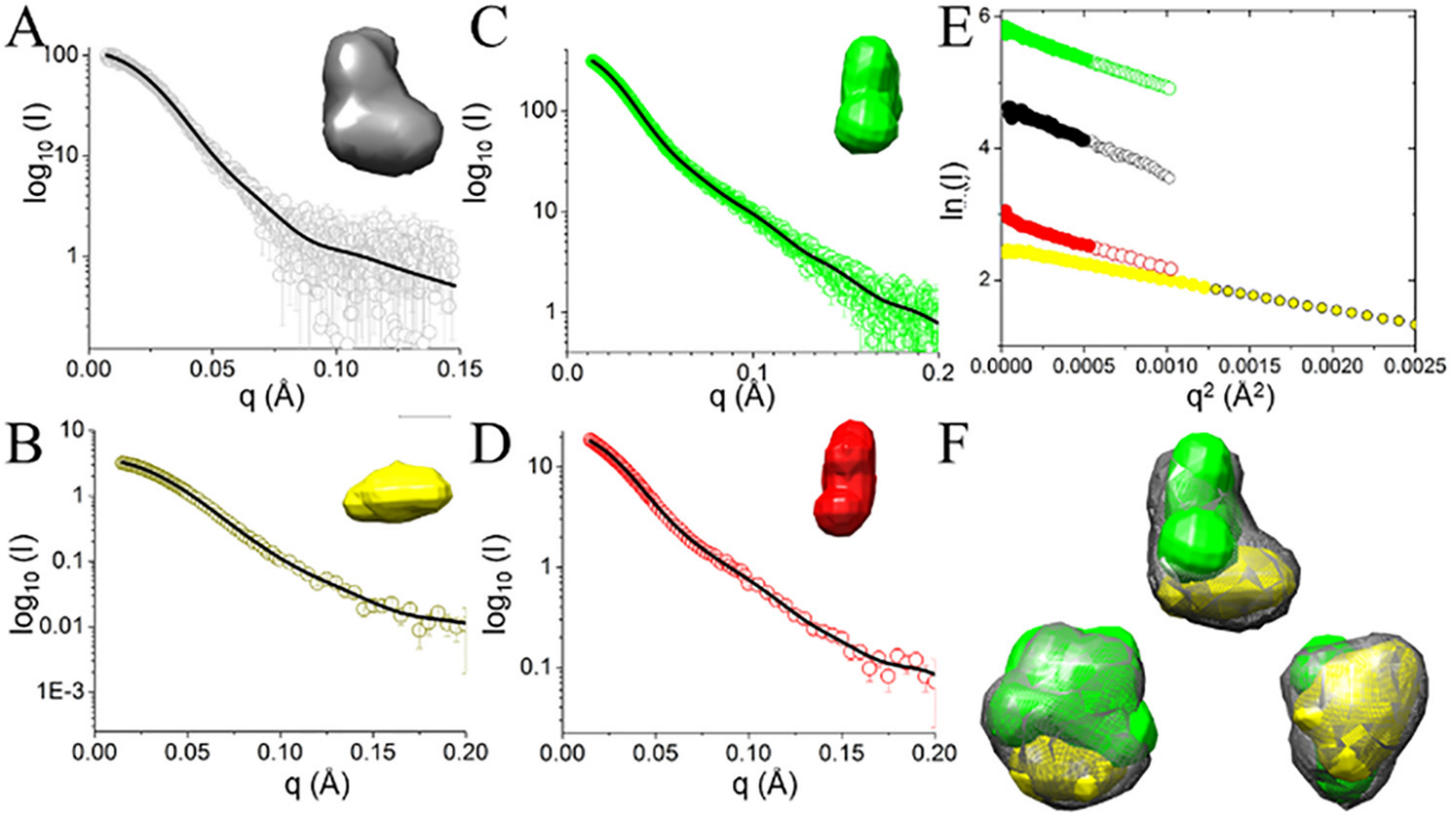
**SAXS analysis indicates that purified VAR2CSA constructs are asymmetric and compactly folded.***A–D*, experimental scattering intensities (*open circles*) and calculated fit (*black lines*) as a function of the momentum transfer (*q*) for constructs with a single view of the averaged *ab initio* envelope (*inset*) (*A*) NTS-DBL6ε, *black*; (*B*) DBL1x-ID2a *yellow*; (*C*) DBL3x-DBL6ε, *green*; (*D*) DBL4ε-DBL6ε *red*. *E,* Guinier plot showing the fitted region as *large filled circles* (linear fit model; Pearson CC -0.99 for all constructs) and *smaller open circles* indicating data beyond the Guinier region, colored as in *A*–*D*. *F,* three orthogonal views of the *ab initio* envelopes of NTS-DBL6ε (*gray mesh*) reveal an asymmetric molecule with a larger broad base and smaller, narrower top. *Ab initio* envelopes for DBL1x-ID2a (*yellow*) and DBL3x-DBL6ε (*green*) show that the envelopes of the two deletion constructs account for the NTS-DBL6ε envelope within the limits of the resolution. For each construct, the averaged/filtered *ab initio* bead models were converted to a pseudo surfaces using Chimera (54).

To gain insight into the structural organization of the ectodomain, NTS-DBL6ε and the deletion constructs, DBL1x-ID2a, DBL3x-DBL6ε, and DBL4ε-DBL6ε, were characterized using SEC-SAXS (Table S3). The data were processed using the ATSAS Suite 3.0 (49, 50) and the values were obtained from this analysis compared with any published results (Table 1, Fig. 2, *A–D*, and Fig. S3). All of the constructs are linear in the Guinier region of the SAXS curve over the given q*R_g_* ranges (Fig. 2*E*), indicating that they are monodisperse. The *R_g_* value obtained for NTS-DBL6ε is smaller than those reported previously for DBL1x-DBL6ε (38, 39) and the *R_g_* value obtained for DBL1x-ID2a is smaller than that reported previously (39), which is consistent with the removal of small amounts of dimer and higher order aggregates during chromatography and the absence of *N*-glycosylation in our constructs. It should be noted that, although the exact domain boundaries are slightly different (Fig. 1*A*), this modification alone would not account for the observed differences in *R_g_* values. For each construct, assessment of the molecular weight (Table 1) using a consensus Bayesian method, implemented in ATSAS 3.0 (51), is in excellent agreement with both theoretical value and those obtained by SDS-PAGE, providing further support that each of the proteins exist as a monomer in solution. For NTS-DBL6ε and DBL1x-ID2a, the pair-distribution, *P*(*r*), function approaches zero smoothly at *r* = 0 Å and the *D*_max_ and has a single peak consistent with relatively globular molecules (Fig. S3A). By contrast, the *P*(*r*) distributions for DBL3x-6ε and DBL4ε-DBL6ε each contain an asymmetric peak with a maxima at ∼42 Å and a shoulder centered at ∼62 Å, indicative of a molecule that contains spatially distinct modules, with the first peak due to the distribution of scattering centers (chords) within a module and the shoulder reflecting largely the chords between the modules. The dimensionless Kratky plots further support this analysis (Fig. S3B). NTS-DBL6ε and DBL1x-ID2a have similar plots that contain a single peak at q*R_g_* = ∼1.7 and (q*R_g_*)^2^I(q)/*I*(0) = 1.1, in agreement with theoretical values of 1.75 and 1.1 for a globular protein (52), respectively, and returning to zero at q*R_g_* ∼5 (Fig. S3B). The location of the peak is consistent with each construct behaving in solution as a single module. By contrast, the plots for the DBL3x-DBL6ε and DBL4ε-DBL6ε contain peaks that are shifted to longer q*R_g_* (1.8-1.95), indicative of a more elongated molecule. In addition, the plots for DBL3x-DBL6ε and DBL4ε-DBL6ε contain a shoulder at q*R_g_* ∼ 4.9 and q*R_g_* ∼4, respectively, and return to zero at q*Rg* ∼10, characteristic of proteins containing spatially distinct modules (53).

**Table 1:** Data processing statistics from SAXS measurements

	*R_g_*	q*R_g_* Range for Guinier	Io^a^	*D*_max_ from *P*(*r*)	Molecular mass estimate^b^,^c^	Credibility interval^c^	Nsd (S.D.)	χ^2^ CorMap*p* value	Ambiguity measure^d^
	*Å*	*Å*^2^		*Å*	*kDa*	*Da*	*Å*		
NTS-DBL6ε	55	0.5-1.3	104.1 (0.7)	177	318 (86%)	[221050, 372700] (100%)	0.81 (0.03)	1.1 (0.1)	1.18 (5)
DBL1x-DBL6ε^e^	62	NP^f^		185	NP	NA	NP	NA	NA
DBL1x-DBL6ε^g^	63	0.8-1.3		219	360/431^h^	NA	0.72-0.8 (NP)	1.1 (NA)	NA
DBL3x-DBL6ε	53	0.4-1.3	338.5 (1.0)	160	208 (84%)	[176600, 221050] (100%)	0.95 (0.37)	0.9 (0.4)	2.31 (5)
DBL4ε-DBL6ε	49	0.6-1.3	22.1 (0.1)	148	157 (68%)	[151450, 176600] (99%)	0.98 (0.04)	0.8 (0.1)	2.42 (5)
DBL1x-ID2a	38	0.4-1.3	12.1 (0.3)	132	94 (37%)	[77400, 99200] (91%)	0.99 (0.28)	0.5 (0.5)	1.57 (5)
DBL1x-ID2a^g^	48	0.9-1.3		166	167/111^h^	NA	0.72-0.8 (NP)	2.5 (NA)	NA
DBL1x-DBL4ε^g^	55	0.8-1.3		188	307/229^h^	NA	0.72-0.8 (NP)	1.5 (NA)	NA
ID1-DBL4ε^g^	49	0.6-1.3		169	207/183^h^	NA	0.72-0.8 (NP)	1.3 (NA)	NA


a Number in parentheses are S.D.

b Numbers in parentheses are probability estimates of the molecular mass.

c Described in Ref. 51.

d Described in Ref. 78.

e Published in Ref. 38.

f NP, not published; NA, not available at the time of their publication.

g Published in Ref. 39.

h The first number is the molecular weight derived from the SAXS curve and the second number calculated from the cloned protein sequence.

### Ab initio bead model of VAR2CSA constructs reveal their overall shape

For each construct, an *ab initio* averaged/filtered bead model was calculated from the models of 20 individual simulations with DAMMIF in the ATSAS Suite 3.0. The simulated SAXS curves for these models are in good agreement with the data, as judged by the χ2 values (Table 1). For NTS-DBL6ε, the ensemble of individual calculated models is similar (Table 1), resulting in an asymmetric bead model of approximate dimensions 160 Å × 130 Å × 90 Å. It has a thinner, ∼50 Å wide head in its narrowest dimension and a wider base of ∼90 Å (Fig. 2*A*). This is in good agreement with the approximate dimensions of crystal structures of DBL domains; for example, the crystal structure of DBL6ε (42) is an elongated molecule of approximate dimensions 35 Å × 45 Å × 85 Å (calculated *R_g_* 23 Å) that can be accommodated within either the head or the base.

To identify the location of DBL1x-ID2a, DBL3x-DBL6ε, and DBL4ε-DBL6ε within NTS-DBL6ε, *ab initio* bead models were calculated as before. For DBL1x-ID2a, the averaged bead model is smaller than NTS-DBL6ε, as expected from its smaller molecular weight, with approximate dimensions of 115 Å × 75 Å × 50 Å (Fig. 2*B*). For DBL3x-DBL6ε, the averaged model is asymmetric and elongated in shape (135 Å × 100 Å × 75 Å) (Fig. 2*C*). For DBL4ε-DBL6ε, the consensus bead model is a triangular prism reminiscent of a letter T (165 Å × 100 Å × 75 Å) with the vertical leg ∼50 Å in diameter and the horizontal arm ∼115 Å × 65 Å × 45 Å (Fig. 2*D*).

The bead models of the fragments were then fit into NTS-DBL6ε using chimera, placing DBL3x-DBL6ε first because it was the largest fragment. Although individually, each could occupy a range of positions, there was only one arrangement in which DBL3x-DBL6ε and DBL1x-ID2a fit into the full-length envelope simultaneously without a large conformational rearrangement (Fig. 2*F*). DBL4ε-DBL6ε fits inside DBL3x-DBL6ε leaving an additional volume that, presumably, corresponds to DBL3x; however, it could not be placed in a unique orientation. Thus, the SAXS data are consistent with DBL1x-2IDa occupying the base of the molecule and DBL3x-DBL6ε occupying the length of the molecule including a thinner head region.

### EM analysis of negatively stained single particles reveal the spatial arrangements of DBL domains in VAR2CSA

**Figure 3:**
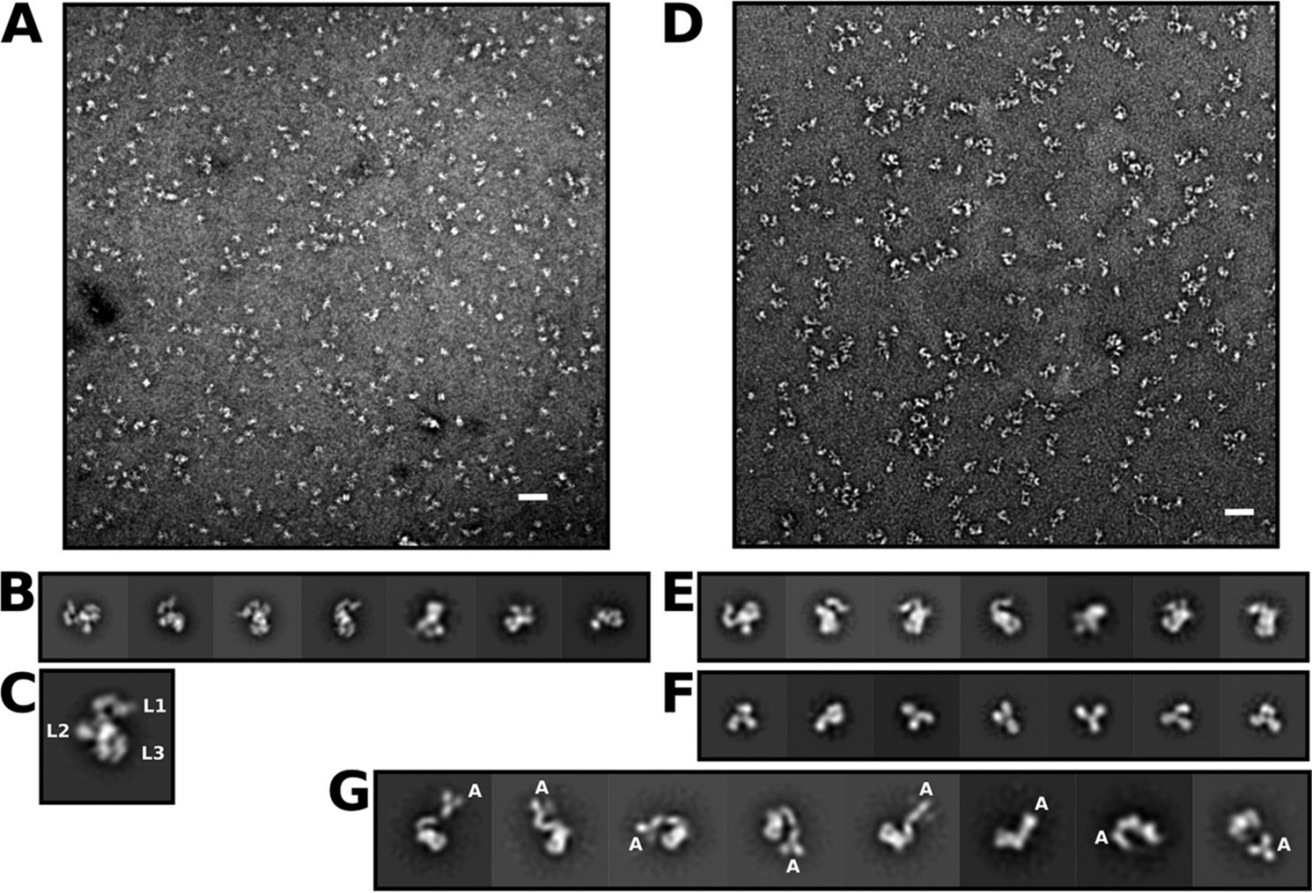
**VAR2CSA is an asymmetric molecule with the C terminus located within the smaller lobe.***A,* micrograph of NTS-DBL6ε. The *white bar* is 50 nm. *B,* subset of 2D classes obtained during initial 2D classification of NTS-DBL6ε. *C,* single 2D class showing the layers (L1, L2, L3), separated by *broken white lines*, defined in the text. *D,* micrograph of NTS-DBL6ε bound to anti-cMyc antibody. *E,* representative 2D classes of unbound NTS-DBL6ε. *F,* representative 2D classes of unbound anti-cMyc antibody. *G,* representative 2D classes of NTS-DBL6ε:anti-cMyc-antibody complex. The letter *A* indicates the location of the cMyc antibody at the tip of the smaller lobe of NTS-DBL6ε.

To generate a higher resolution model than possible by SEC-SAXS analysis, single particle reconstruction of negative-stained images was calculated for all of the constructs. During data collection, examination of regions throughout the grids revealed that all constructs produced both positively and negatively stained images on the same grid and, in some cases, within the same field. Therefore, data were collected from areas of the grids in which negatively stained particles dominated. Fig. 3*A* shows a representative negative-stained micrograph with well-isolated NTS-DBL6ε particles. 2D classification of particles picked from this and other equivalent images (Fig. 3*B* and Fig. S4) gives a range of well-defined classes that, in side view, suggests a roughly rectangular particle with three layers of stain-excluded density (L1-3) arranged into two asymmetrically-distributed lobes (Fig. 3*C*). The smaller lobe (L1) contains a single distinct volume and is referred to hereafter as the head, whereas the larger lobe (L2, L3) is comprised of three distinct regions, hereafter called the body, the tail, and the feet. Furthermore, inspection of these side-on classes suggest that the head is connected to the body via a thin, stain-excluding neck (*red arrows* in Fig. S4). A second, smaller stain-excluding volume from the middle of the head to the body (*green arrows* in Fig. S4) may also be a direct connection or, alternatively, result from a superposition of distinct structural features in some projections. The location of the C terminus of DBL6ε in the structure was identified in negative-stain images of NTS-DBL6ε bound to an anti-cMyc IgG type mAb (Fig. 3*D*). Particles were picked from this and equivalent images to obtain 2D classes that fit into one of three distinct subsets: classes similar to those observed with NTS-DBL6ε alone (Fig. 3*E*); Y-shaped classes in projection that are characteristic views for the free mAb (Fig. 3*F*); and classes corresponding to side-on images of complexes between NTS-DBL6ε and the Y-shaped mAb (Fig. 3*G*). In this final set of 2D classes, the Y-shaped mAb is clearly visible at the end of the head, providing an unambiguous identification of the location of the C-terminal cMyc tag adjacent to the head domain (small lobe). It follows that DBL6ε is located in the density adjacent to the bound antibody at the free end of the head, furthest away from the neck and body. The relatively short linker between DBL5ε and DBL6ε (∼10-20 amino acids) and the clear connectivity of the density in the head suggests the remaining head density arises from DBL5ε.

**Figure 4:**
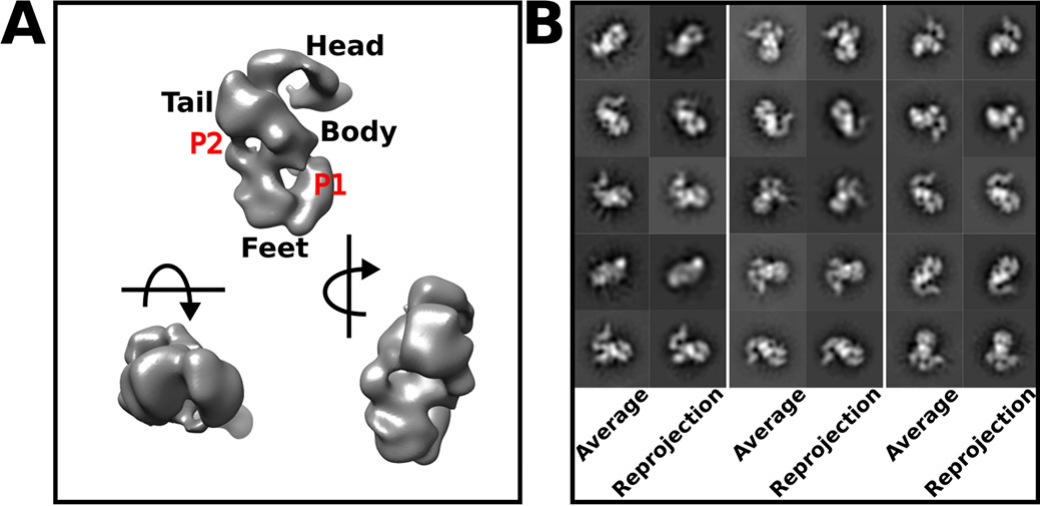
**The NTS-DBL6ε reconstructed volume contains distinct regions and is consistent with the 2D classes.***A,* three orthogonal views of the 3D reconstructed NTS-DBL6ε molecule with head, tail, body, and feet, as indicated. Two pores P1 and P2 run through the protein. The *arrows* show the direction of rotation relative to the *top left view*. *B,* the top 15 2D classes, representing ≥90% of the particles used in the 3D reconstruction, paired with their corresponding back projection of the 3D model. All 3D volumes in this and subsequent figures were visualized using Chimera (74).

Particles selected from the best 2D classes were used to reconstruct the 3D volume of NTS-DBL6ε (Fig. 4*A* and Table 2). This reconstruction shows that the ectodomain adopts a slightly extended conformation with overall dimensions, 175 Å × 160 Å × 110 Å (Fig. 4*A*), which are slightly larger than those estimated from the corresponding SAXS bead model. Consistent with the views in the 2D classes, the 3D volume is comprised of three layers that are together broadly reminiscent of a duck, with a head, body, feet, and tail (Fig. 4*A*). The strong concordance between the reprojections from the 3D volumes and their corresponding 2D classes (Fig. 4*B* and Table 2) provides validation for the NTS-DBL6ε model. The first layer comprises the head (125 Å × 75 Å × 65 Å) and is of sufficient volume to accommodate two DBL domains. The middle layer has a larger volume comprising the body (100 Å × 80 Å × 60 Å) and bulbous DBL domain-sized tail (65 Å × 60 Å × 55 Å) that protrudes from it. The final layer is a C-shaped volume (120 Å × 85 Å × 60 Å) comprising the feet; a central cleft (20 Å × 30 Å × 95 Å) separates each DBL sized foot.

**Table 2:** Data processing statistics for single particle reconstruction of negative-stained EM

	NTS-DBL6ε	DBL1x-ID2a	ID2b-DBL6ε	DBL3x-DBL6ε	DBL4ε-DBL6ε
No. micrographs	184	150	45	80	60
CTF range (μm)	∼0.8-2.0	0.6-1.0	0.8-1.6	0.8-1.6	0.8-1.6
No. particles picked^a^	35,256	29,614	22,095	31,888	30,430
No. particles 2D classification^b^	24,127	13,305	7,723	18,871	11,233
No. particles in final model^c^	9,822	10,260	4,763	17,423	8,032
Reprojection cost final model^d^	0.91-0.98	0.93-0.98	0.90-0.95	0.86-0.96	0.83-0.95
Gold standard FSC (gsFSC)^e^	22 Å	22 Å	28 Å	25 Å	25 Å


a Particle set picked with Relion 3.1 Lorentizan of Gaussian autopick routine.

b Particles following 2-3 rounds of 2D classification in Relion that were used to start 3D reconstruction.

c Particles set used to calculate the final refined model.

d Range of xmipp_cost parameter for reprojection analysis 3D model and 2D classes representing ≥90% of the particles used the final reconstruction.

e Reported resolution from Relion 3.1 post-processing.

**Figure 5:**
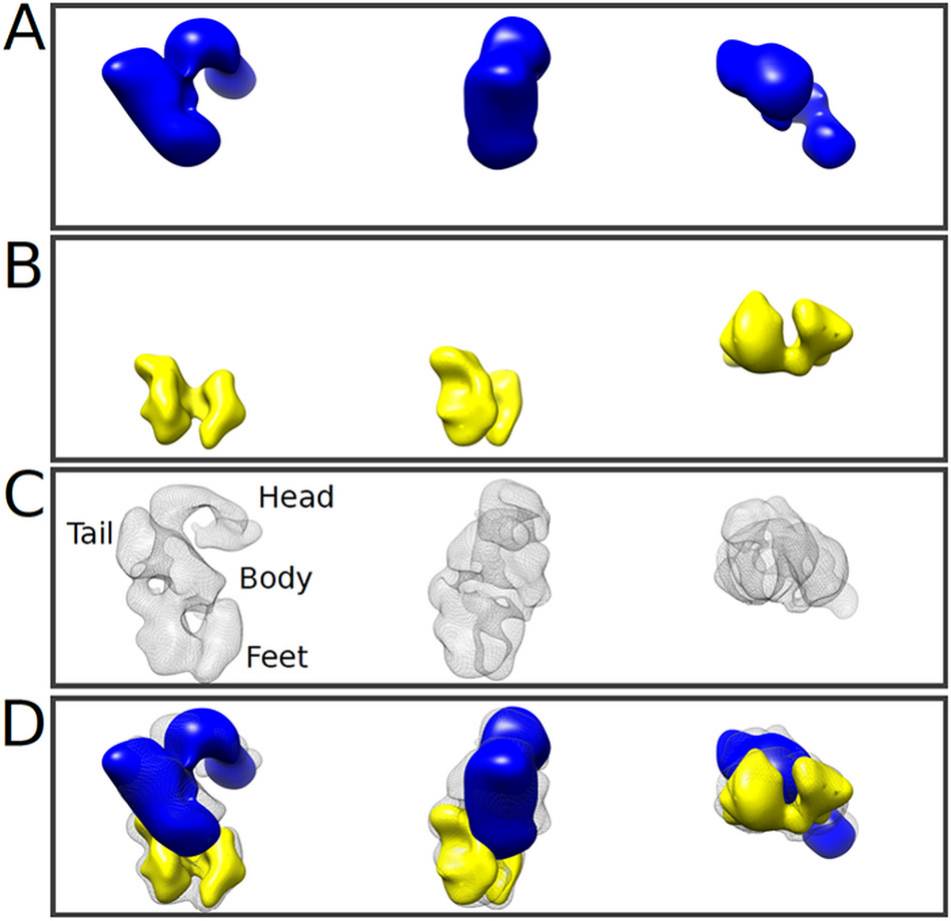
**The reconstructions delineate the locations of the DBL domains.** Three orthogonal views of the reconstructed volume of (*A*) ID2b-DBL6ε (*blue solid*), (*B*) DBL1x-ID2a (*yellow solid*), and (*C*) NTS-DBL6ε (*black mesh*). *D, A*–*C* superimposed. ID2b-DBL6ε, forms the head and tail of the molecule and DBL1x-ID2a the feet. ID2 appears to form a bridge linking DBL1x and DBL2x to the remainder of the molecule.

To determine whether the particular structural features in layers L1–L3 of NTS-DBL6ε could be assigned to specific regions of the protein, single particle reconstructions of negative-stained EM images of the N- and C-terminal deletion constructs ID2b-DBL6ε and DBL1x-2IDa, respectively, were calculated and the resulting volumes compared with the EM-based reconstruction of NTS-DBL6ε. For the N-terminal deletion construct ID2b-DBL6ε (Fig. 5*A* and Fig. S5), an S-shaped molecule containing two weak stain-excluding regions was visible in some 2D classes (denoted by *red* and *green arrows* in Fig. S5A) strongly resembling the L1 and L2 regions observed in 2D classes of NTS-DBL6ε. This S-shaped arrangement also defined the 3D volume, which is comprised of two cylindrical arms ∼60 Å in diameter and 120 Å in length (Fig. 5*A*) and had similar characteristics to the head, tail, and body volumes of NTS-DBL6ε. For the C-terminal deletion construct, DBL1x-ID2a, an asymmetric bilobal molecule, was visible in 2D classes (Fig. S6) most strongly resembling the L3 region observed in the 2D classes of NTS-DBL6ε. The corresponding reconstructed volume is globular (125 × 85 × 80 Å) with a distinct cleft ∼20 Å in diameter (Fig. 5*B* and Fig. S6), which is also visible in the feet of the NTS-DBL6ε volume. Attempts to identify the location the C-terminal cMyc tag in the DBL1x-2IDa volume were unsuccessful (Fig. S7) preventing assignment of specific densities to the individual DBL1x, DBL2x, and 2IDa domains. The assignment of 3D features was confirmed by superposition of the DBL1x-ID2a and ID2b-DBL6ε volumes into that of NTS-DBL6ε (Fig. 5, *C* and *D*). As expected from our analysis, the DBL1x-ID2a volume localizes to the “feet” and ID2b-DBL6ε to the head, body, and tail features of the NTS-DBL6ε volume, respectively. Together, these data demonstrate that L1 contains DBL5ε and DBL6ε, L2 contains DBL3x and DBL4ε, and L3 contains DBL1x, ID1, and DBL2x. Placed in this manner, the tip of the larger density in the DBL1x-ID2a reconstruction extends from the feet into the region in the body near the DBL3x-DBL4ε volume. This suggests that DBL2x and ID2a would be contained in the larger volume and thus DBL1x in the smaller volume of the DBL1x-2IDa reconstruction.

**Figure 6:**
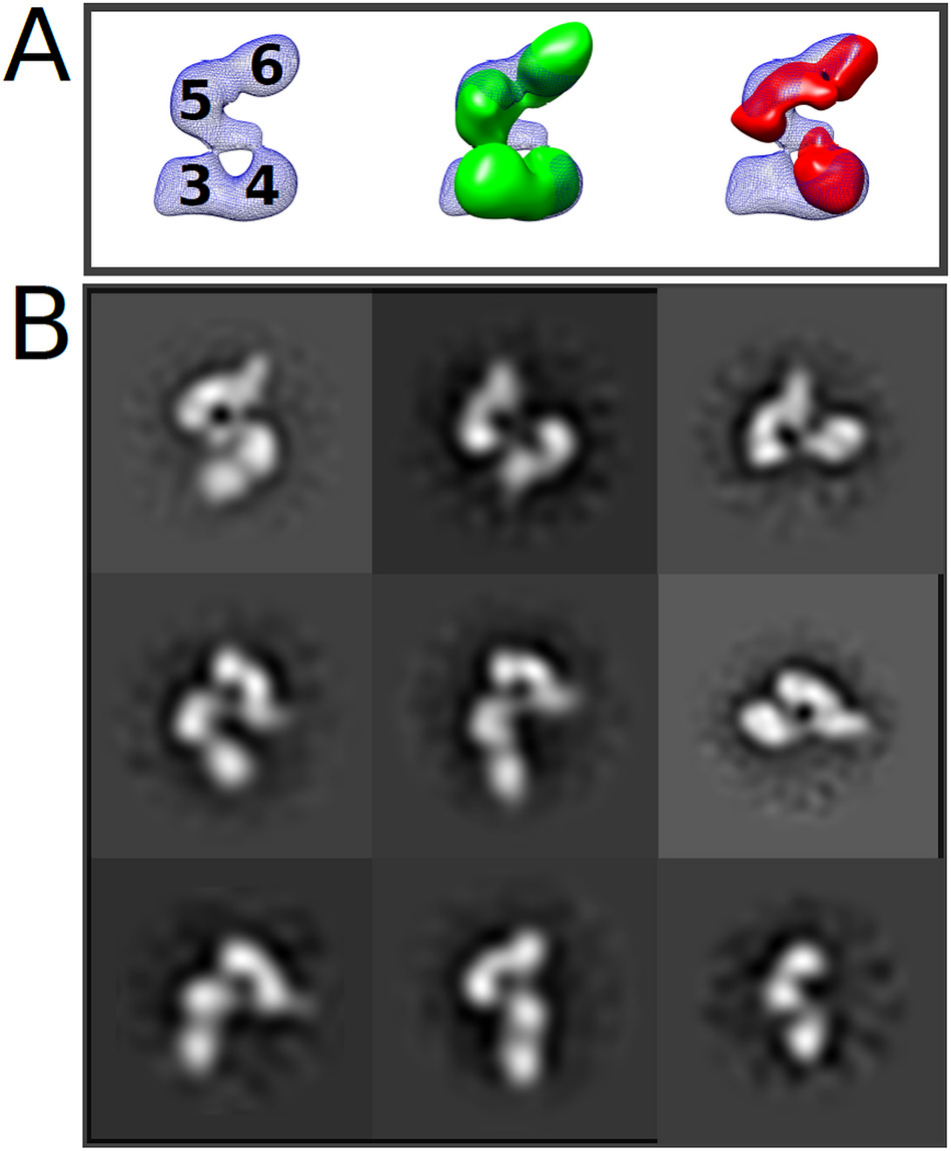
**Superposition of the three N-terminal deletion constructs allows identification of DBL3x (3), DBL4ε (4), DBL5ε (5), and DBL6ε (6) to defined volumes in ID2b-DBL6ε.***A,* DBL2b-DBL6ε (*blue mesh; left panel*) is used as the reference to place DBL3x-DBL6ε (*green solid; middle panel*) and DBL4ε-DBL6ε (*red solid; right panel*). *B,* placement of domains is consistent with features of 2D projections as featured in three equivalent 2D classes for DBL2b-DBL6ε (*left column*), DBL3x-DBL6ε (*middle column*), and DBL4ε-DBL6ε (*right column*).

To further assign volumes in L2, single particle reconstructions of DBL3x-DBL6ε (Fig. S8) and DBL4ε-DBL6ε (Fig. S9) were calculated and aligned with that of ID2b-DBL6ε (Fig. 6*A*). The DBL3x-DBL6ε construct (Fig. 6*A* and Fig. S8) is an S-shaped molecule (approximate dimensions 160 Å × 135 Å × 65 Å) with four clear, stain-excluding regions in both 2D classes and in the 3D-reconstructed volume. The DBL4ε-DBL6ε construct is a skewed T-shaped molecule (approximate dimensions 150 Å × 130 Å × 65 Å), with 3 clear stain-excluding volumes, which is evident in both the 2D classes and in the 3D map (Table 2, Fig. 6*A*, and Fig. S9). The relatively weaker stain-excluding densities connecting L1 and L2 observed in ID2b-DBL6ε are less striking in the DBL3x-DBL6ε and DBL4ε-DBL6ε reconstructions; however, corresponding maps and projections are consistent with the observed 2D classes (Figs. S8 and S9). The weaker connecting density is likely due to a combination of a lower signal-to-noise ratio of images of these constructs and, potentially, relatively more conformational freedom (Table 2). The volumes of DBL3x-DBL6ε and DBL4ε-DBL6ε were fit into that of ID2b-DBL6ε using an automated fitting algorithm in Chimera (Fig. 6*A*). Because the distinctive volume of DBL5ε and DBL6ε in L1 is common to all N-terminal deletion constructs, it provided a visual constrain for assessing fit. In each case, applying this constrain, the placement of volumes is unique and reveals that in L2, DBL4ε is located below both DBL5ε and DBL6ε in the body and DBL3x forms the tail (Fig. 6*A*). It should be noted that for all constructs containing DBL4ε, DBL5ε, and DBL6ε, the domains occupy equivalent locations in all maps, however, in the DBL3x-DBL6ε map, the volume corresponding to DBL6ε is rotated by ∼45° relative to its location in NTS-DBL6ε and ID2b-DBL6ε. The distinctly different orientation of the terminal domain is also evident when comparing some 2D classes (Fig. 6*B*, *bottom panel*). Furthermore, although the two arms of the S-shape are better defined in the volume of ID2b-DBL6ε (Fig. 6*A*), there was no obvious connected density that could be assigned to ID2b. It is not clear whether the presence of ID2b directly improves the reconstruction quality via a stabilization of the structure or it is simply due to the higher signal-to-noise of the negative-stained images for this construct.

The structural analysis of the EM and SAXS data were performed independently and thus a comparison of the models obtained from each technique provides reliable validation for the proposed structure. Adjusted for resolution, the volumes for each of the constructs generated by SAXS and negative-stained EM are in good agreement. To further compare the models, EM density maps were converted to dummy atom models using EM2DAM (ATSAS 3.0) and theoretical SAXS curves were calculated from them and compared with the experimental SAXS curves (Fig. S10). There was good agreement between these curves and, as expected, the EM-based curve contained additional features consistent with finer detail and less movement or degeneracy.

### Docking of experimentally and computationally derived domain modules into the EM reconstructions and comparison to NTS-DBL6ε SAXS data

**Figure 7:**
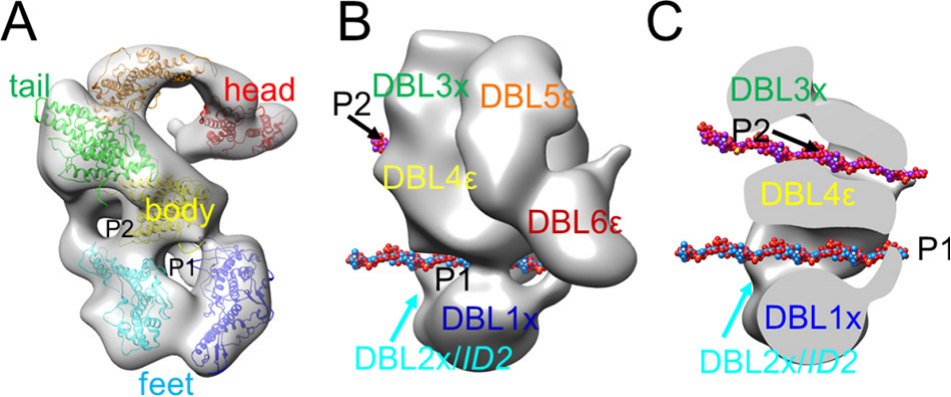
**Placement of DBL1x-DBL6ε, based on the set of EM reconstructions, suggests a possible model for carbohydrate binding.***A,* semi-transparent surface of the NTS-DBL6ε map (*gray*), into which homology models of DBL1x (*blue*), DBL2x (*cyan*), DBL3x (*green*), DBL4ε (*yellow*), DBL5ε (*orange*), and DBL6ε (*red*) have been positioned, reveals that 2 pores, P1 and P2, are formed by DBL1x-DBL4ε. Models for individual domains and the templates used are detailed in Table S4. *B,* solid representation of NTS-DBL6ε map showing a 12-mer CSA (PDB code 1CSA), in sphere representation, positioned in each pore. Carbon atoms are colored *light blue* and *purple* in pore P1 and P2, respectively. Oxygen, nitrogen, and sulfur atoms are colored *red*, *blue,* and *yellow*, respectively. *C,* transverse section through the NTS-DBL6ε with domains labeled as in *B*.

For rigid body fitting of data, models corresponding to DBL1x, DBL2x, DBL4ε, and DBL5ε were generating using Chimera and Modeller (54, 55). Structures of DBL3x and DBL6ε were used directly after modeling regions that were disordered in the crystal structures using the Chimera Modeller loops/refinement plug-in (55). However, ID1 (∼200 amino acids) and ID2 (∼300 amino acids) could not be modeled as suitable structural templates are not available (Table S4). The domains were placed as shown in Fig. 7*A.* DBL6ε fit into the expected density at the tip of the molecule and DBL5ε fit into the remaining density in the head. Based on the connection determined in the negative strain reconstruction, DBL3x fit into the tail and DBL4ε was constrained to be adjacent to DBL3x and DBL5ε. DBL1x and DBL2x fit into the base, although the arrangement of these two domains is speculative because the folds of ID1 and ID2 are not known. DBL3x and DBL4ε can be accommodated in an arrangement similar to the structure of the DBL3x-DBL4ε tandem domains (44). The DBL domains account for ∼75% of the ectodomain and ID domains and linker sequences accounting for the remainder of the molecule. Excluding the ID domains, the average length of the linkers is 40 residues. These two factors make placement of individual domains into low resolution SAXS envelopes degenerate without using constraints determined from other models (such as fixing the position of DBL6ε using information derived from EM). However, the placement of DBL domains determined from the EM reconstructions is in good agreement with the SAXS curve (Fig. S10E). Fitting the domains as described, reveals two large pores, P1 and P2, in NTS-DBL6ε that are created by DBL1x, DBL2x, and DBL4ε (and likely ID1), and DBL2x, DBL3x and DBL4ε, respectively (Fig. 7, *B* and *C*).

## Discussion

The VAR2CSA ectodomain and the N- and C-terminal deletion constructs described in this work are produced in a mammalian expression system that exports them into the media as folded proteins. Because Pf does not possess the cellular machinery to add *N*-glycosylation moieties to nascent polypeptides post-translationally, potential *N*-glycosylation sites in the protein sequence, which might be modified during export in the HEK 293F cells, were removed by site-directed mutagenesis, as described by Srivastava *et al.* (38). The *O-*glycosylation content of the proteins was not characterized. The purified proteins are very stable, as expected due to the presence of a large number of intra-domain disulfide bonds. Trypsin digestion of purified protein followed by LC–MS/MS resulted in the identification of 15 phosphorylated residues (Fig. S10, Table S4, and in the PRIDE repository). These included the three phosphoresidues important for *in vitro* adhesion, which are present in both recombinant DBL1x-DBL6ε and Pf expressed VAR2CSA on the surface of infected red blood cells (56), providing further support that these constructs are correctly folded. Each construct remained soluble and monomeric at concentrations >5 mg/ml, demonstrating their suitability for structural characterization. Analysis by SAXS and negative-stain EM show that the protein constructs are homogeneous in terms of overall shape and apparent sizes. Taken together, we contend that the structures discussed here largely represent authentically folded VAR2CSA and folded domains thereof. Additionally, the *ab initio* envelope of NTS-DBL6ε calculated from SEC-SAXS, described here, contains the same features as published models after making allowances for the presence of the estimated 5% glycosylation in protein produced from the baculovirus expression system (38, 39). This suggests that slight differences in constructs and data collection/processing do not affect the overall structure of the molecule.

The single particle EM reconstructions of the ectodomain of VAR2CSA define the overall duck-like architecture of the molecule. Comparison of the SEC-SAXS bead models and negative-stained EM reconstructions of the intact ectodomain and their various deletion constructs clearly define the locations of the DBL3x through DBL6ε domains and reveal the location of the DBL1x-ID2a module within this structure both in solution and on grids. The observed connectivity and overall structural details observed in the EM and SAXS data presented here reveals a packing of three tandem domains: DBL1x with DBL2x, DBL3x with DBL4ε, and DBL5ε with DBL6ε. This packing is distinctly different from the SAXS-based model proposed by Clausen *et al.* (39) that comprised of three tightly packed domain pairs DBL1x with DBL6ε, DBL2x with DBL5ε, and DBL3x with DBL4ε and result in the N- and C-terminal domains co-localizing.

Structurally, both the SAXS envelopes and negative-stain reconstructions suggest that the intact ectodomain, DBL1x-ID2a, and, to a somewhat lesser extent, DBL3x-DBL6ε and DBL4ε-DBL6ε are structurally rigid at this resolution despite the paucity of obvious contacts between DBL5ε-DBL6ε and the body and tail of the structures (DBL3x, DBL4ε, and some portion of ID2). Examination of the disulfide bonding pattern observed in the previously determined crystal structures identifies 8 canonical intra-domain disulfide bonds, although only one set (C(8)-C(12)) is observed in all the crystal structures of DBL3x (PDG codes 3CML and 3BQI), DBL4ε (part of PDB code 4P1T), and DBL6ε (PDB codes 2WAU and 2Y8D). However, the remaining surface-exposed, free cysteine residues potentially are available to form inter-domain disulfide bonds that could stabilize the overall architecture of VAR2CSA. Although the current studies, due to their limited resolution, do not provide direct evidence for the existence of inter-domain disulfide bonds, at least two discrete volumes of stain-excluding density between the spatially distinct regions of the head and DBL4ε are observed in 2D classes of all DBL4ε-containing reconstructions. This is consistent with the presence of inter disulfide bonds and could account, in part, for the observed rigidity of this region of the structure. It should be noted that only one of these densities, which we term the neck, connecting one end of the head to DBL4ε is routinely observed in the 3D maps at the typical contour levels used. This most likely reflects a combination of conformational heterogeneity between the head and the body and limitations in image quality and number for the minor tilted views compared with the dominant side-on views. Despite being rigid, overall the structure is relatively open, which would allow access of kinases to the various domains that are phosphorylated in the endogenous and recombinant VAR2CSA (56). The two phosphorylated residues in DBL4ε are contained at the beginning of the domain within a long surface-exposed loop that runs the width of the domain that, in its current position, would also be surface exposed in the context of the full-length protein. The phosphorylation site in DBL5ε is located in helix 7 that is one of the two long helices in the DBL domain (42). The remaining phosphosites are located within ID1 and ID2, for which there are no convincing templates, however, the disk-shaped density for DBL1x-ID2a suggest that there is a large solvent accessible surface area.

A striking characteristic of the NTS-DBL6ε structure is the presence of two pores, P1 and P2, which are approximately parallel and traverse the width of the protein, accounting for the layers seen in some 2D classes. Based on the structure of CSA (PDB code 1C4S) each pore has dimensions that could accommodate CSA 10-12-mers (Fig. 7, *B* and *C*). In this model, binding of carbohydrate in P1 would involve residues from DBL1x, ID1, DBL2x, DBL4ε, and likely ID2a, and in P2 involve residues from DBL2x, DBL3x, DBL4ε, and possibly ID2b. Enveloping the carbohydrate in a pore provides a larger surface area for binding interactions while minimizing the accessibility of this functionally-essential surface to immune surveillance. CSA and its nonsulfated form, chondroitin, can be described as stiff worm like polymers (persistence lengths ∼90-110 Å (57)). Based on the structure of CSA (PDB code 1CSA), a fully extended 12-mer CSA chain has an end-to-end distance of ∼100-115 Å with an estimated minimum root mean square end-to-end distance of ∼90-100 Å, although it is likely to be closer to the fully extended length in solution (58). Therefore this single 12-mer chain cannot occupy both pores simultaneously without either large scale rearrangements in the highly disulfide bond-stabilized protein or substantial protein-induced hairpin bending of the CSA; either scenario seems unlikely. Many studies have concluded that residues within DBL1x-2IDa are necessary and sufficient for high affinity CSA binding, and the arrangement of domains forming P1 is consistent with these data. Binding studies presented here also show that DBL1x-2IDa has a lower affinity for CSA than NTS-DBL6ε, consistent with involvement of interactions outside of this region, such as DBL4ε in our model. The potential role of P2 in carbohydrate binding, if any, is less clear. One possibility is that it could be involved in protein–protein interactions either in the knob or during transport from the parasite to the surface of the red blood cell. A second possibility is that it is a carbohydrate-binding site, although to date, binding studies have not revealed the presence of two high affinity CSA-binding sites. Thus, if each binds a distinct carbohydrate chain, then the second site would have to be much lower affinity than the first and binding would have to be independent. Biologically, this might have some advantage for parasite attachment to the placental extracellular matrix. The CSPG molecules are part of a complex mixture of polymers that form the host extracellular matrix of the intervillous spaces. In the context of this complex host receptor environment, two binding sites on VAR2CSA may facilitate its attachment to a heterogeneous carbohydrate-dense environment. However, at the current resolution it is impossible to clearly determine which of these possibilities, if any, are correct.

**Figure 8:**
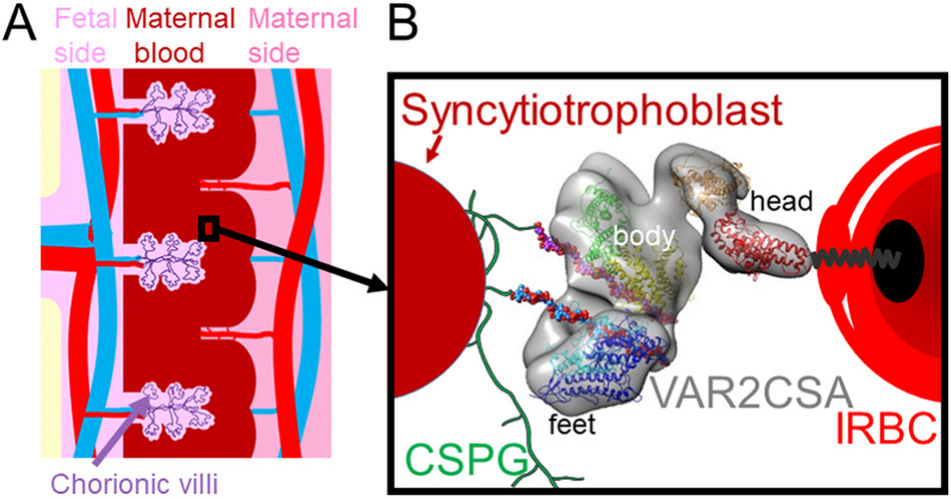
**Schematic diagram illustrating VAR2CSA, expressed on the surface of the IRBC, binding bind to the CSA chains of CSPG receptor in the placenta.***A,* shows the placental vasculature on the fetal (*light pink, the left portion*) and maternal (*dark pink, the right portion*) sides and the villous syncytiotrophoblast in the maternal blood (*brown, middle portion*). The *black box* encloses a region of the intervillous space filled with maternal blood, bathing the syncytiotrophoblast surface. *B,* more detailed diagram of the region enclosed by the *black box in A*. VAR2CSA expressed on the knob membrane of an IRBC (*red*) is anchored by a single transmembrane helix C-terminal (*black*) linking its cytosolic domain (*black oval*) to its ectodomain (*gray*). DBL6ε of the ectodomain is located in the head region closest to the IRBC surface and the surface containing the body and feet (DBL1x-DBL4ε) and pores P1 and P2 is extended toward the syncytiotrophoblast surface containing the CSPG receptor (*green*). The CSA 12-mers that form part of the CSA chains, are shown as *solid spheres*. The structures are not drawn to scale. The DBL domains and CSA molecules are colored according to Fig. 7.

The EM reconstruction of the ectodomain, described here, provides additional insight into how VAR2CSA might function in the context of infected red blood cells binding to CSA. Previous studies have shown that, in the knob-like structures observed on the surface of infected cells, VAR2CSA molecules are present largely at the tips (59, 60). The identification of the C terminus of the ectodomain with the anti-cMyc mAb constrains the location of DBL6ε to be adjacent to the IRBC membrane. Building on these data, and the fact that the molecule is rigid, we can speculate about the orientation of the DBL domains in the intervillous space of the placenta (Fig. 8). In this schematic diagram, the head of VAR2CSA ectodomain sits closest to the erythrocyte membrane, whereas the body and feet are located at the membrane-distal tip of the structure extending out from the knob. Anchoring the head allows relatively free access of the body and feet to bind the placental CSA in the intervillous space. We have speculated that the pores facilitate receptor binding, however, the general scheme also allows for CSA binding within the NTS-DBL1x-ID1-DBL2x-ID2a region that is required for specific binding of the CSA ligand (61). By contrast, if DBL1x and DBL6ε were adjacent, as suggested in the previous model for domain organization (62), then the putative CSA-binding domains (DBL1x-DBL2x) would be located nearer to the knob membrane, making it less accessible to the placental CSA chains. The proposed scheme and observed rigidity also suggests how atypical VAR2CSA, expressed by placental parasite isolates that encode one or two additional C-terminal domains (DBL7ε and DBL8ε) (63, 64) could be accommodated without interfering with the putative CSA-binding surface. The additional domains would, presumably, insert between the “head” and the transmembrane helix, either forming a larger head or extending the body and feet further from the IRBC surface (Fig. 8). Although this work focuses on placental malaria, the domain organization is also conserved in nonplacental, non-CSA binding PfEMP1 genes that bind different receptors, such as ICAM1, endothelial protein C receptor, and CD36 that sequester the IRBCs in other organs (65). The receptor-binding sites of these PfEMP1s are hydrophobic and located in the first half of their sequences (66, 67, 68). Therefore, the model presented here might be relevant to the wider PfEMP1 family because the arrangement orients the receptor-binding sites away from the IRBC membrane.

In conclusion, these studies have revealed for the first time, the structure of VAR2CSA, at moderate resolution, its orientation relative to the IRBC membrane and the relative arrangement of domains. The structure contains two clear pores that transverse the molecule and suggest a possible model for host receptor binding. This model provides plausible explanations for the high affinity CSA interaction and the ability to evade initial immune surveillance.

## Experimental procedures

### Synthesis and subcloning of VAR2CSA gene into pSecTag2-based expression vectors

The sequence of the codon-optimized VAR2CSA gene from strain 3D7 (JQ247428.1) was used as a starting point to generate a synthetic gene (amino acids 59-2641). Serine or threonine residues at 20 sites, identified as sites of potential *N-*linked glycosylation arising from the mammalian machinery (38), were replaced with codons for alanine or leucine (GeneArt, ThermoFisher Scientific). KpnI and ApaI sites were added to the 5′ and 3′ end, respectively, for subcloning into the pSecTag2/Hygro2 vector, which contains a N-terminal IgK secretion signal and noncleavable C-terminal cMyc and His_6_ tags for protein purification. In the constructs encoding ID2b-DBL6ε and DBL4ε-DBL6ε, a cMyc and FLAG-tagged versions were created. The cMyc tag in the vector was replaced by a TEV-cleavable 3× FLAG tag using a GBLOCK primer listed in Table S1. The cMyc-tagged versions of these constructs were used for carbohydrate binding studies only. To generate NTS-DBL6ε, a GBLOCK (IDT) (Table S1) corresponding to amino acids 1-58 was designed and inserted into pSecTag2/Hygro A vector using In Fusion (Takara, Japan) according to the manufacturer's protocol to create a vector that contained amino acids 1-58 followed by KpnI and ApaI sites. Residues 59-2641 were then subcloned as before, which generated insertion of a glycine and threonine residue between native residues 58 and 59. DBL1x-ID2a (aa 59-1019), ID2b-DBL6ε (aa 1016-2641), DBL3x-DBL6ε (aa 1218-2630), and DBL4ε-DBL6ε (aa 1560-2630) were created by PCR using the plasmid DNA of DBL1x-DBL6ε (1 ng/μl) as a template, primers (Table S1) and the Q5 site-directed mutagenesis kit (New England Biolabs) for polymerase and parental DNA digestion. All DNA constructs were sequenced through the coding region to confirm that the sequences were correct (Eurofins Genomics, Lancaster, PA). The translated protein sequences of the constructs are detailed in Table S2.

### Protein expression and purification

The expression and purification of proteins is based on Srivatsava *et al.* (44) with minor modifications. HEK 293F cells were grown in FreeStyle 293 serum-free expression medium (Invitrogen catalog number 12338-018) and the relevant expression plasmid was transfected using polyethyleneimine, at a ratio of 1:3, DNA:polyethyleneimine, and grown at 37 °C. After 24 h, cells were diluted with an equal volume of Freestyle 293 medium, prewarmed to 37 °C, and supplemented with 2.2 mm valproic acid (catalog number P4543-100G, Sigma). In some cases, protease inhibitors (catalog number K1008, Apexbio Technology Cat; final concentration 1× from a 200× stock) were added to the media after 48 h and the cultures were harvested 72 h post-transfection, by centrifugation at 6000 × *g*, 4 °C. The clarified media, which contained the exported VAR2CSA proteins, was filtered through a 0.22-μm filter, protease inhibitor mixture (200 × in DMSO, Apexbio Technology, K1008) added to a final concentration of 1× and the media either frozen at −20 °C or used directly for protein purification. Proteins were purified from fresh or thawed media by a concentration to 50 ml using a VivaFlow 50 cross-flow cassette MWCO 100 k (catalog number VF05C4, Sartorius), diluted 1:1 with 20 mm imidazole in Ni buffer (500 mm NaCl, 10 mm NaKHPO_4_, pH 7.0), then reconcentrated to a final volume of 50 ml. The expressed proteins were isolated via a three-step procedure. Processed medium was applied to a HisTrap Fast Flow HPLC column (catalog number 17531901, GE Healthcare), pre-equilibrated with Ni Buffer containing 20 mm imidazole and washed with 5 column volumes of Ni Buffer containing 35 mm imidazole. The bound protein was eluted in a step gradient with Ni Buffer containing 300 mm imidazole. The peak fractions containing the VAR2CSA protein were concentrated using a Centricon 100-kDa concentrator and exchanged to CPX Buffer A (50 mm NaCl in 10 mm NaKHPO_4_, pH 6.8) prior to ion-exchange chromatography. The concentrated protein was loaded on an Eshmuno CPX column (8 × 20 mm, Sigma) pre-equilibrated with CPX Buffer A. The column was washed with 5 column volumes of 95% 50 mm CPX Buffer A:5% CPX Buffer B (1 m NaCl in 10 mm NaKHPO_4_, pH 6.8) and the bound protein was eluted in a 10-column volume linear gradient from 40 to 80% CPX Buffer B. The peak fractions were analyzed by SDS-PAGE and Coomassie Blue staining. Protein-containing fractions were pooled and concentrated (Microsep, 100 kDa cutoff) to ∼5 mg/ml and applied to a TSK-G3000SWxL column (7.8 × 30 cm, Tosoh Bioscience, King of Prussia, PA) pre-equilibrated with SEC Buffer (150 mm NaCl in 10 mm NaKHPO_4_, pH 6.8). The protein was eluted with SEC Buffer and protein-containing fractions were analyzed by SDS-PAGE and Coomassie Blue and/or silver staining. Fractions containing purified proteins (>95% pure) were concentrated to 2-8 mg/ml. The proteins were either used for experiments immediately or snap frozen in liquid nitrogen and stored at −80 °C for future use.

### CD measurements

CD spectra (190-260 nm) of proteins (0.2 mg/ml) in 10 mm NaKHPO_4_, pH 6.8, 100 mm NaF were recorded on a Jasco J-1500 spectrophotometer at 25 and 90 °C using a 0.1-cm path length cuvette. Spectra were accumulated at 0.5-nm data intervals at a scan rate of 50 nm/min. For each sample, three scans were collected, averaged, and corrected for base line rotation. Data were converted to molar ellipticity. For thermal denaturation, spectra of proteins (0.2 mg/ml) in 0.1 m NaCl and 10 mm NaKHPO_4_, pH 6.8, were recorded using a Jasco J-720 spectrophotometer at 222 nm over the temperature range 25 to 85 °C at 1 °C intervals. Data were processed using GraphPad Prism, version 5.

### Analysis of phosphorylation by MS

The phosphorylation sites in NTS-DBL6ε were determined by in-gel tryptic digestion followed by LC–MS/MS of the resultant peptides (56, 69, 70). NTS-DBL6ε (2 μg) was separated by 4–20% SDS-PAGE under reducing conditions and visualized using Coomassie Blue staining (56). The band corresponding to NTS-DBL6ε was excised and transferred to a 1.5-ml microcentrifuge tube containing 50 μl of 25 mm ammonium bicarbonate, pH 8.3. The cysteine sulfhydryl groups were reduced by addition of 5 μl of 10 mm DTT and incubation at 56 °C for 30 min followed by addition of 5 μl of 55 mm iodoacetamide and further incubation for 20 min at room temperature in the dark. The protein was digested using 60 ng of trypsin (1:30 trypsin:NTS-DBL6ε, w/w; Promega Madison, WI) for 12 h at 37 ˚C (69). Proteolysis was stopped by the addition of 0.5 μl of 98% TFA and precipitates were removed by centrifugation at 12,000 × *g* for 15 min at 4 °C. The supernatant was desalted using C18 Ziptips (Millipore) and eluted in 50% acetonitrile containing 0.5% formic acid and lyophilized. The lyophilized peptides were reconstituted in 10 μl of 3% acetonitrile containing 0.1% formic acid and analyzed by LC–MS/MS. Approximately 0.5 μg of the digested peptides were injected from a NanoLC AS-2 autosampler (Eksigent, Dublin, CA) into a NanoLC-Ultra-2D Plus HPLC (Eksigent, Dublin, CA) using a 10-μl injector loop. Trap and elute mode was used to separate each SCX fraction using the microfluidics on a cHiPLC Nanoflex system equipped with a Trap column (200 μm × 0.5-mm ChromXP 3C18-CL 120 Å resin) and a separation column (75 μm × 15 cm ChromXP 3C18-CL 120 Å resin). The column was subsequently washed with mobile phase A (100% water and 0.1% formic acid, v/v) for 1 min at a flow rate of 0.5 μl/min. The peptides were eluted in a linear gradient from 95% A:5% B (100% acetonitrile: 0.1% formic acid) to 70% A:30% B at 0.3 μl/min over 50 min. The eluted peptides were analyzed by in-line detection with an AB Sciex 5600+ Triple TOF MS/MS (AB Sciex, Framingham, MA) mass spectrometer operated in positive and high sensitivity modes. For data acquisition, the detector was set to a mass range of 400 to 1250 (*m*/*z*), charge state range of 2+ to 6+, an accumulation time of 250 ms, a collision energy of 10 V, a declustering potential of 80 V, an ion spray potential of 25500 V, and an interface heater temperature of 150 °C. Peptide fragmentation was accomplished by collision-induced dissociation using nitrogen at a pressure of 3.6 × 105 Torr. A maximum of 50 parent ions were subjected to fragmentation per cycle. Fragmentation spectra were collected over a mass range of 50-6000 Da with an accumulation time of 25 ms and rolling collision energy, as determined automatically by the embedded software, and collision energy spread of 3 V. The peptides were identified using Protein Pilot version 5.0.1 with embedded Paragon algorithm version 5.0.1.0, 4874 (71, 72) against the NTS-DBL6ε sequence database. For peptide identification the precursor mass tolerance was 5 ppm, the fragment mass tolerance at 50 ppm, and a false discovery rate of 1%. Carbamidomethylation (+57.0215 Da) and methionine oxidation (+15.9949 Da) were set as fixed modifications. The identified peptides were included in the results if they met all the following criteria: score >10, confidence >99%, peak elution within 30 s, peak intensity 1000 counts. The peptides were manually matched to the NTS-DBL6ε. Phosphopeptides were identified by including a phosphorylation (+79.9663 Da) as a variable modification in the search parameters and an intensity cutoff of >500 counts. Phosphorylation sites were confirmed manually, by identifying the phosphosite-determining *b* and *y* ions in the spectra.

### Analysis of VAR2CSA proteins binding to CSPG

The extent of binding of the VAR2CSA proteins was assessed in a sandwich ELISA-based assay using bovine corneal CSPG-II (73). The preparation of bovine corneal CSPG-II used here contains low-sulfated CSA chains with 28% 4-sulfated, 3% 6-sulfated, and 69% nonsulfated disaccharide moieties. Briefly, wells of a 96-well–microtiter plate were coated with bovine corneal CSPG-II by incubation with 50 μl (200 ng/ml of CSPG-II in PBS, pH 7.2) at 4 °C overnight. All subsequent steps of this assay were performed at room temperature. Unbound CSPG-II was removed by washing the wells with 100 μl of PBS, pH 7.2, containing 0.05% Tween-20 (PBST) and then all wells were blocked with 100 μl of 1% BSA in PBS, pH 7.2, for 2 h to minimize nonspecific binding. Binding of the individual VAR2CSA proteins was assessed by serial 2-fold dilutions (50 μl) of the cMyc-tagged VAR2CSA construct (see Fig. S2) into CSPG-II–coated and uncoated (blank, to assess nonspecific protein binding) wells of the microtiter plate and incubated for 2 h. Unbound protein was removed by washing 3 times with 100 μl of PBST. 50 μl of 1:1000 diluted mouse anti-cMyc mAb (cMyc mAb, catalog number NB600-302, NOVUS Biologicals, Centennial, CO) was then added to each well, washed three times with 100 μl of PBST, incubated with 50 μl of 1:10,000 diluted HRP-conjugated goat anti-mouse IgG (Jackson ImmunoResearch, West Grove, PA), and then washed three times with 100 μl of PBST. The bound cMyc mAb was quantitated by the addition of 50 μl of 3,3‘,5,5‘-tetramethylbenzidine substrate and the color was allowed to develop for 20 min at room temperature to ensure that the assays were in the linear detection range. Color development was stopped by addition of 25 μl of 2.5 m HCl and the absorbance at 450 nm measured to estimate the extent of protein bound to the CSPG-coated wells.

### Inhibition of VAR2CSA proteins binding to CSPG by various glycosaminoglycans (GAGs)

A modified version of the binding assay, outlined above, was used to analyze the ability of GAGs to inhibit binding of NTS-DBL6ε and DBL1x-ID2a to CSPG-II. The following GAGs were evaluated: bovine tracheal CSA (54% 4-sulfated, 39% 6-sulfated, and 8% nonsulfated disaccharide moieties; catalog number C-8529, Sigma), CSC (∼20% 4-sulfated and ∼80% 6-sulfated disaccharide moieties, Seikagaku Corp, Japan), and HA (100% nonsulfated disaccharide moieties; catalog number H-1504, Sigma). Microtiter plates were coated with bovine corneal CSPG-II, described above. Separately, NTS-DBL6ε and DBL1x-ID2a proteins (2 μg/ml in PBS, pH 7.2) were incubated at room temperature with GAGs at concentrations ranging from 0.03 to 5 μg/ml. After 40 min, 50-μl solutions of protein:GAG mixtures were added to the CSPG-coated plates and incubated for 1 h. The extent of protein binding to the CSPG-coated wells was measured as described above.

### Small angle X-ray scattering measurements

Data were collected by in-line SEC-SAXS either at LiX 16-ID beamline of the National Synchrotron Light Source II (NSLSII), Brookhaven National Laboratory, Upton, NY or 18ID of the Advanced Photon Source (APS), Argonne National Laboratory, Argonne, IL (Table 1, Table S3). For data collection, 250 μl of each sample was applied to a Superose 6 10/300 GL size-exclusion column (GE Healthcare) at concentrations between 4 and 10 mg/ml and eluted at room temperature with a flow rate of 0.5 ml/min in 10 mm NaKHPO_4_, pH 6.8, 150 mm NaCl. Scattering curves were collected continuously throughout the elution to obtain buffer and protein scattering curves. Data were initially processed using beamline software py4XS (NSLSII) and RAW (APS) and subsequently programs in the ATSAS Suite (versions 2.8 and 3.0) (53). *Ab initio* bead models were calculated from 20 candidate models calculated in DAMMIF, averaged using DAMAVER and refined with DAMMIN. For display purposes and superposition, the bead models were converted to volumes within Chimera and superimposed using the fitmap algorithm (54, 55, 74).

### Preparation of NTS-DBL6ε–anti-cMyc antibody complex and DBL1x-ID2a–anti-cMyc antibody complex for EM

cMyc mAb (catalog number NB600-302, Novus Biologicals, CO) and NTS-DBL6ε or DBL1x-ID2a proteins were mixed at a ratio of 1:2 molar ratio and incubated for 30 min on ice. Solutions were loaded onto a TGX3000S size-exclusion column (Tosho Biosciences) and eluted with 150 mm NaCl, 10 mm NaKHPO_4_, pH 6.8, at a flow rate of 0.5 ml/min. Fractions containing protein–antibody complexes were identified by the absorbance profile and SDS-PAGE of individual fractions, pooled, concentrated to 0.5 mg/ml, and used for negative staining and EM.

### Negative staining and EM analysis

Formvar-coated carbon grids (300 μm mesh; EMS, PA) were prepared by plasma cleaning (air) for 60 s using an Emitech K590X plasma cleaner (Diener Electronics, Germany) immediately prior to use. For NTS-DBL6ε, or DBL1x-2IDa, a 4-μl aliquot of the protein alone, or bound to cMyc mAb (25 μg/ml in 10 mm NaKHPO_4_, pH 6.8, 150 mm NaCl) was applied to the grids and incubated at room temperature for 10 s. Excess protein solution was removed from the edge of the grid using thin strips of Whatman No. 1 paper. The grids were washed 3 times with distilled water by placing 35-μl drop and each time touching the surface with filter paper and excess water removed as before. For staining, the grids were floated sequentially on the top of three 35-μl drops of 1% uranyl acetate in water for 10 s for the first two drops and 1 min for the final drop, air dried at room temperature for 1 h, and stored under anhydrous conditions until needed. EM data were collected using a JEOL 2100 transmission electron microscope equipped with a 4K CCD camera. All images were collected at 200 eV at a pixel size of 2.3 Å/pixel using Digital Micrograph.

### Single particle reconstruction from EM micrographs of negative-stain images

Single particle reconstructions were performed in Relion 3.1 (75) and validated using tools and protocols in Scipion 2 (76). Particles from 5 to 10 images were picked automatically using a range of minimum and maximum particle diameters and thresholds with the Lorenztian of Gaussian algorithm to estimate optimal values. Particles were then picked from the entire set of images using the same algorithm and the optimized numbers for each parameter and extracted, using a box size of 1.5 times the longest dimension of the particle, with a pixel size of 6.9 Å to improve signal to noise. The particle stack was subjected to 2-3 rounds of 2D classification to obtain a starting particle set for 3D reconstruction. Initial starting models were computed to 25 Å resolution using a randomly chosen subset of 4000 particles. Subsequently, all of the particles were used in 3D classification with the calculated starting model as a reference. The best class or classes were subjected to 3D refinement and post-processed to obtain a final masked map. The reported resolution was obtained using the gold standard FSC (gsFSC) during post-processing. In some cases, a second round of 2D/3D classification and refinement improved the shape of the gsFSC. In these cases, the particles from the first 3D refinement were subjected to an additional round of 2D classification followed by 3D classification with a single class. The resultant map was further refined and post-processed, as before, to yield the final map described in this work. The maps for all constructs were validated using the 3D reprojection and overfitting tools in Scipion 2 with data from the final refinement in Relion 3.1 to confirm that there was no overfitting within the resolution ranges used. A summary of the results of reconstruction and 3D reprojection are given in Table 2 and Figs. S4–S9.

### Basic homology modeling of individual domains

Templates for homology modeling were identified using the BLAST server (RRID:SCR_004870) and visualized using Chimera. Basic homology modeling was performed using the MODELLER interface within Chimera. For each domain, models were evaluated by visual inspection, and GA341 and zDOPE scores (Table S4). Residues 1-9, 402-568, 906-1204, 1945-1978, and 2284-2395 did not have credible templates and are not included in the final assembly.

## Data availability

The MS proteomics data have been deposited to the ProteomeXchange Consortium via the PRIDE (77) with the data set identifier PXD021873. All remaining data are contained within the document and is available upon request from Dr. John Flanagan, Department of Biochemistry and Molecular Biology, Pennsylvania State University College of Medicine. E-mail: jflanagan@psu.edu.
