# Supplementary material for: Molecular architecture and domain arrangement of the placental malaria protein VAR2CSA suggests a model for carbohydrate binding
Source: J Biol Chem. 2021 Jan 13;295(52):18589–603. doi: 10.1074/jbc.RA120.014676 (PMC7939466; doi:10.1074/jbc.RA120.014676)
Supplement: Supplementary file 1 [file mmc1.zip › 161297_2_supp_608047_qhs76w.pdf]

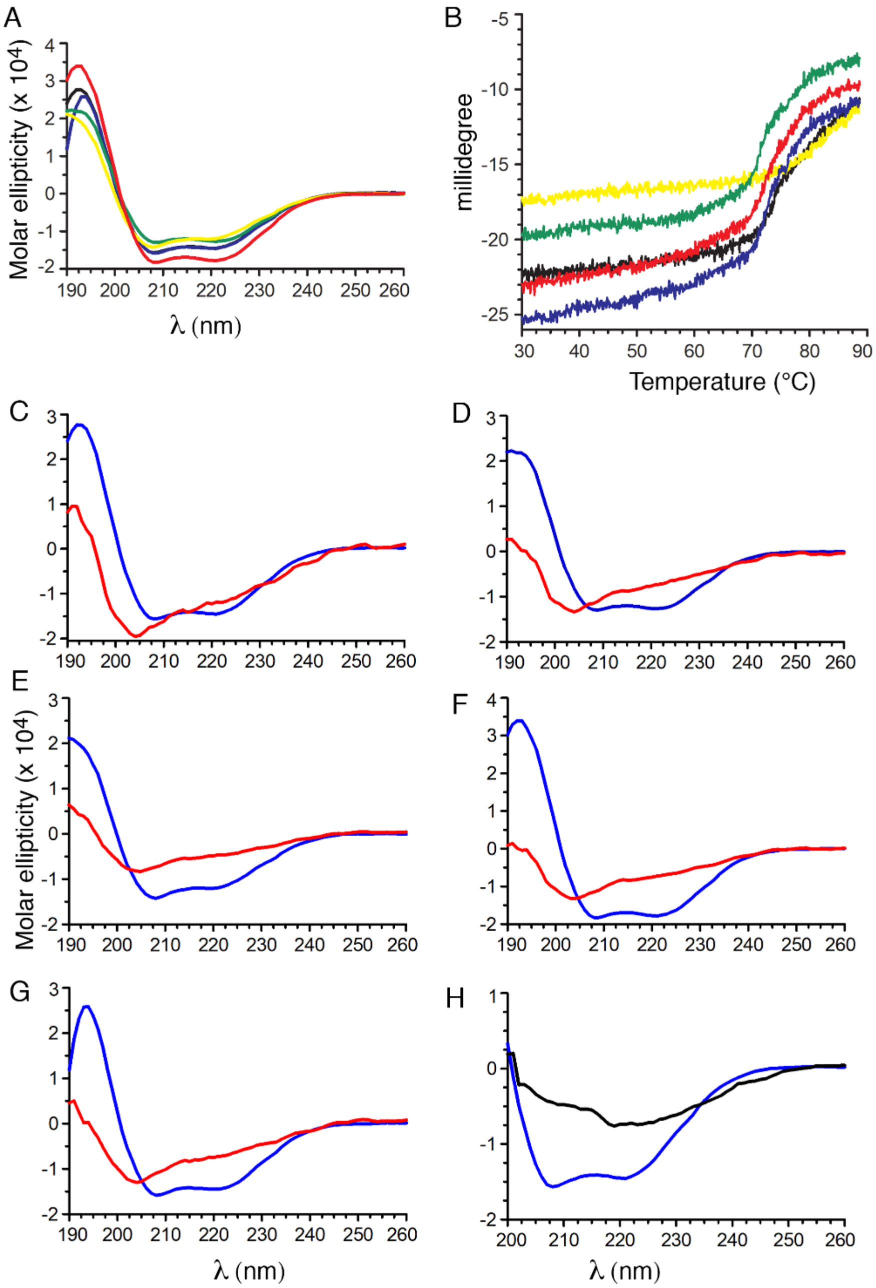

Figure S1. The recombinant VAR2CSA ectodomain and deletion constructs are folded and thermally stable. (A) CD spectra NTS-DBL6ε (black), DBL1x-ID2a (yellow), ID2b-DBL6ε (blue), DBL3x-DBL6ε (green), and DBL4ε-DBL6ε (red) are consistent with proteins containing α-helices. (B) Thermal unfolding curves of the constructs, colored as in (A). (C-G) CD spectra of proteins at 25 °C (blue trace) and 90 °C (red trace) for (C) NTS-DBL6ε, (D) DBL1x-2IDa, (E) ID2b-DBL6ε, (F) DBL3x-DBL6ε and (G) DBL4ε-DBL6ε. (H) NTS-DBL6ε at 25 °C (blue) and NTS-DBL6ε+TCEP at 25 °C (red).

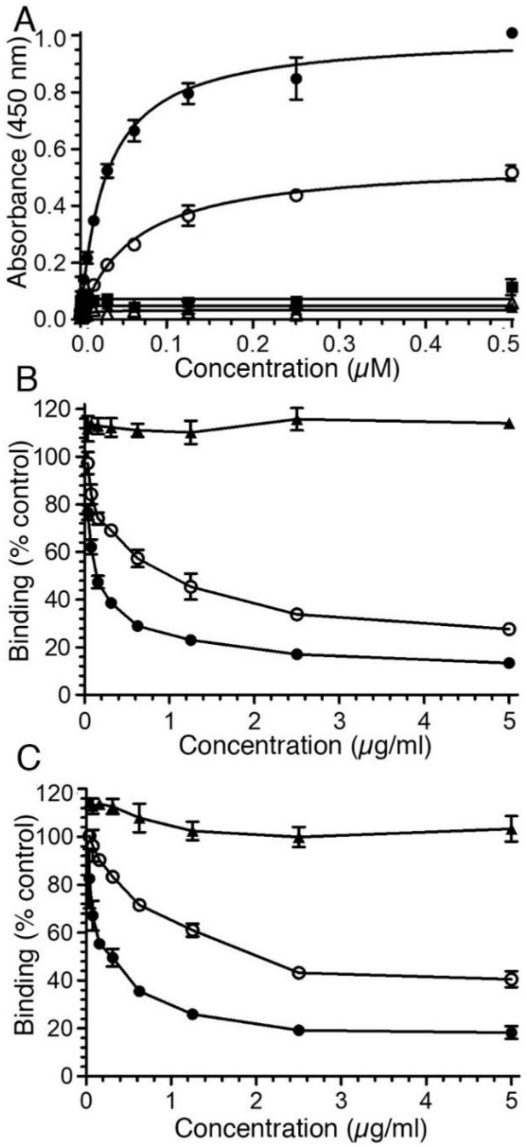

Figure S2. NTS-DBL6ε and DBL1x-2IDa bind C4S. (A) The binding of NTS-DBL6ε and deletion constructs, as described, to CSPG was assessed by an ELISA-based assay. The data were fitted to a non-linear regression 1 site specific binding in PRISM v 7.0. Data points correspond to the following constructs: (●) NTS-DBL6ε; (○) DBL1x-2IDa; (▲) ID2b-DBL6ε; (Δ) DBL3x-DBL6ε; (■) DBL4ε-DBL6ε. (B) Inhibition of NTS-DBL6ε and (C) DBL1x-2IDa binding to CSPG by glycosaminoglycans (●) CSA, (○) C6S and (▲) HA. Error bars represent SD from 3 technical replicates and is representative of three independent analyses.

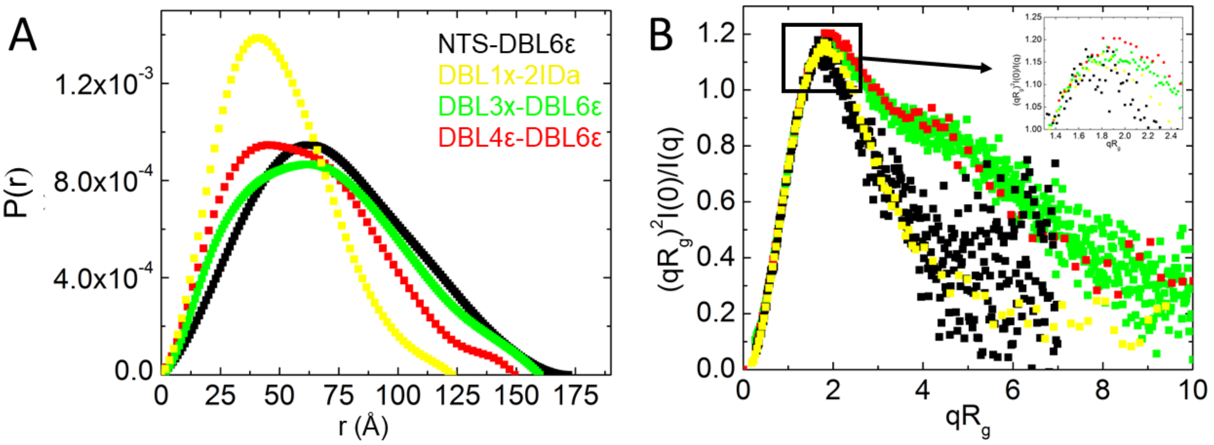

Figure S3 Additional SAXS plots. (A) P(r) plot normalized to area for each of the protein constructs. (B) Dimensionless Kratky plot, colored as in (A) shows a peak at qR_g ~1.7, as expected for folded proteins. DBL3x-DBL6ε and DBL4ε-DBL6ε show an additional shoulder at R_g, typical of protein containing distinct masses connected by a linker. Inset shows a more detailed view of the peak, covering the region shown in the black box.

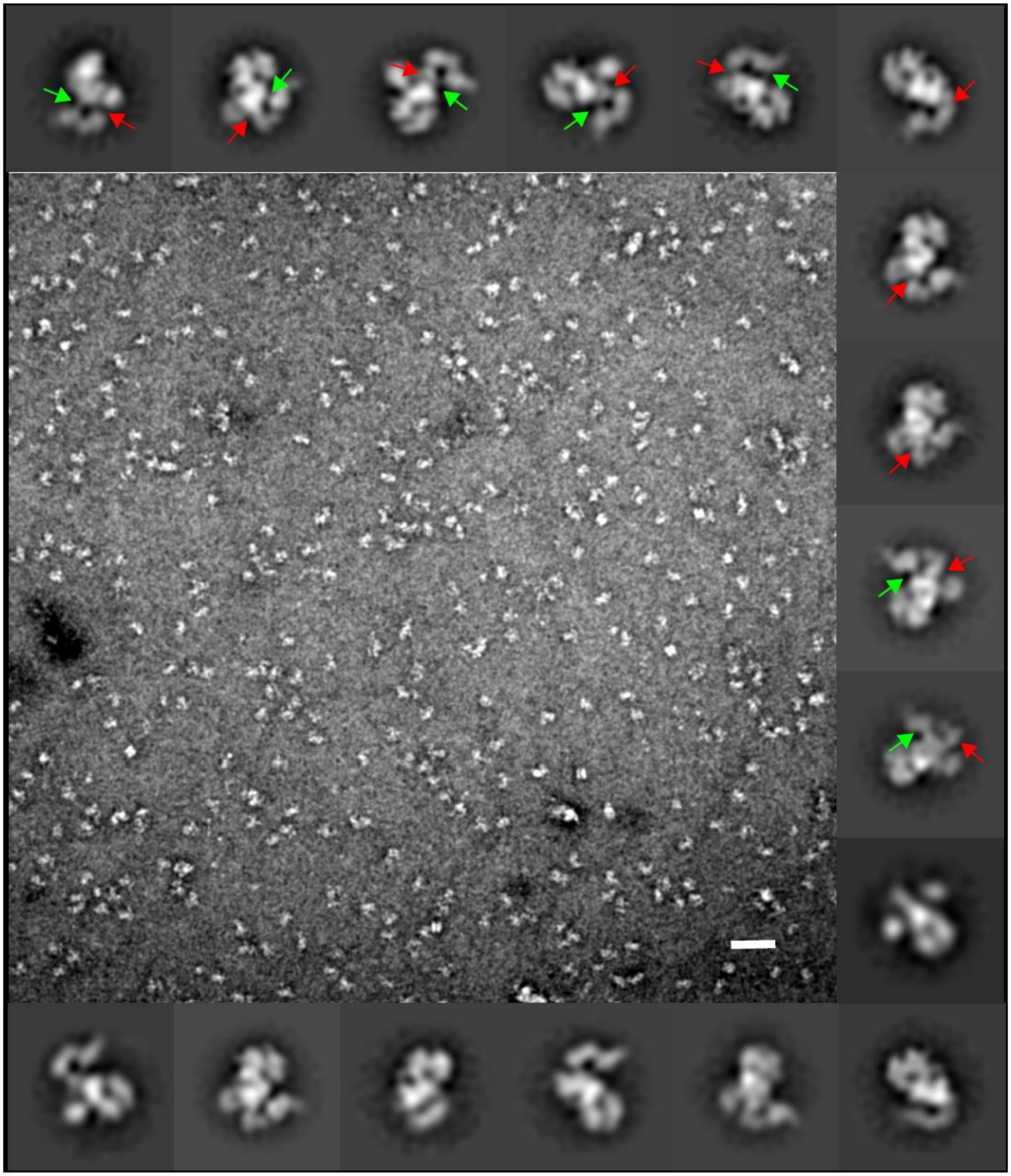

Figure S4. Micrograph and 2D classes of NTS-DBL6ε. (A) Sample micrograph containing particles of uranyl acetate stained NTS-DBL6ε. The white bar is 50 nm. A gallery of 2D classes obtained following the second round of 2D classification, surround the micrograph. Arrows denote the locations of the neck (red) and a second stain-excluding area (green) described in the text.

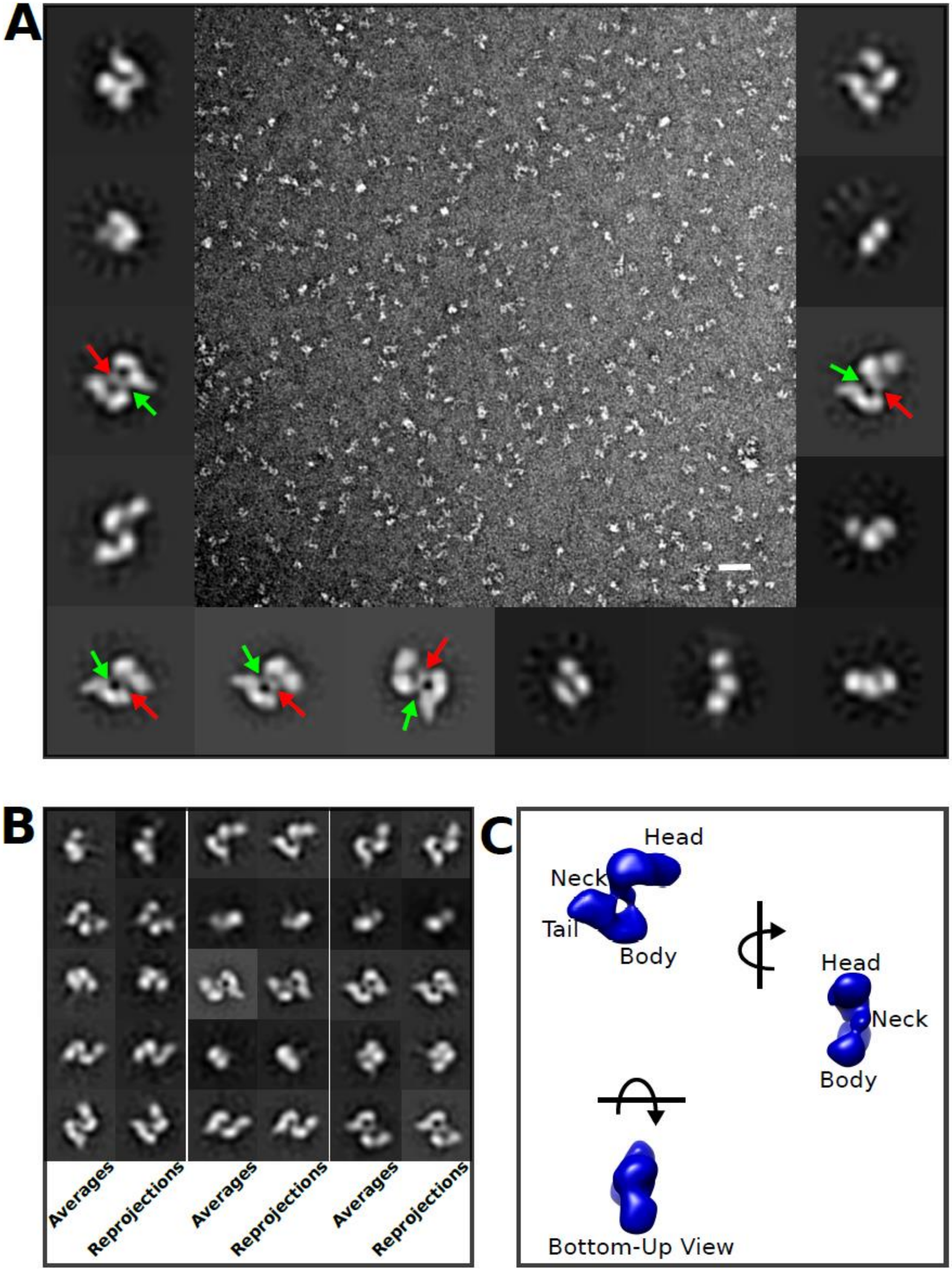
A
B
Averages
Reprojections
Averages
Reprojections
Averages
Reprojections
C
Head
Neck
Tail
Body
Head
Neck
Body
Bottom-Up View

Figure S5. Data and 3-D reconstruction of ID2b-DBL6ε. (A) Sample micrograph containing particles of uranyl acetate stained ID2b-DBL6ε. The white bar is 50 nm. A gallery of 2D classes obtained following the second round of 2D classification, surround the micrograph. (B) The top 15 2D-classes, as defined in Fig. 4, paired with the corresponding back projection of the 3D reconstruction. (C) Orthogonal views of the 3D reconstruction.

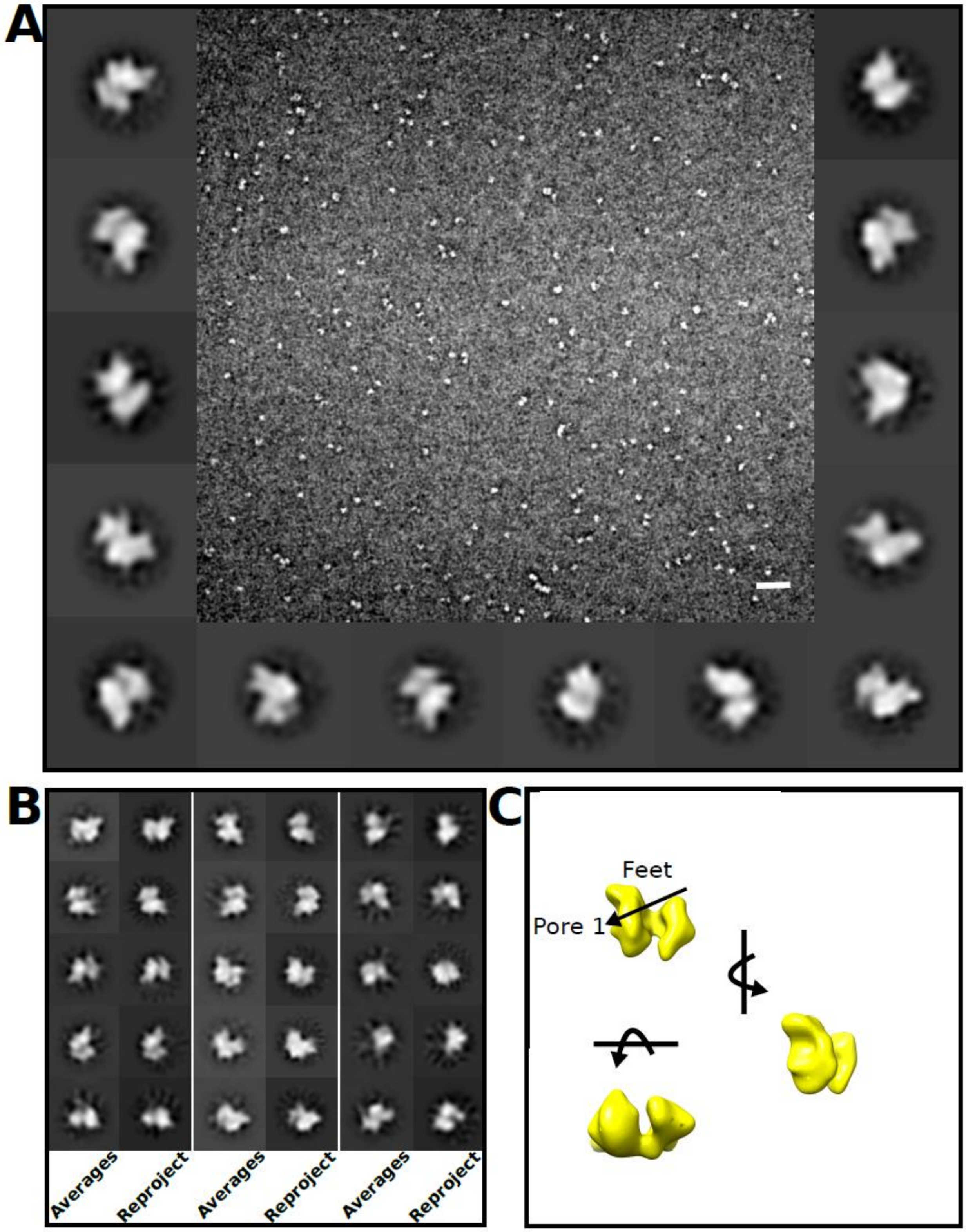

Figure S6. Data and 3-D reconstruction of DBL1x-2IDa. (A) Sample micrograph containing particles of

uranyl acetate stained DBL1x-2IDa. A galley of 2D classes surround the micrograph. (B) The top 25 2D-classes paired with the corresponding back projection of the 3D reconstruction. (C) Orthogonal views of the 3D reconstruction.

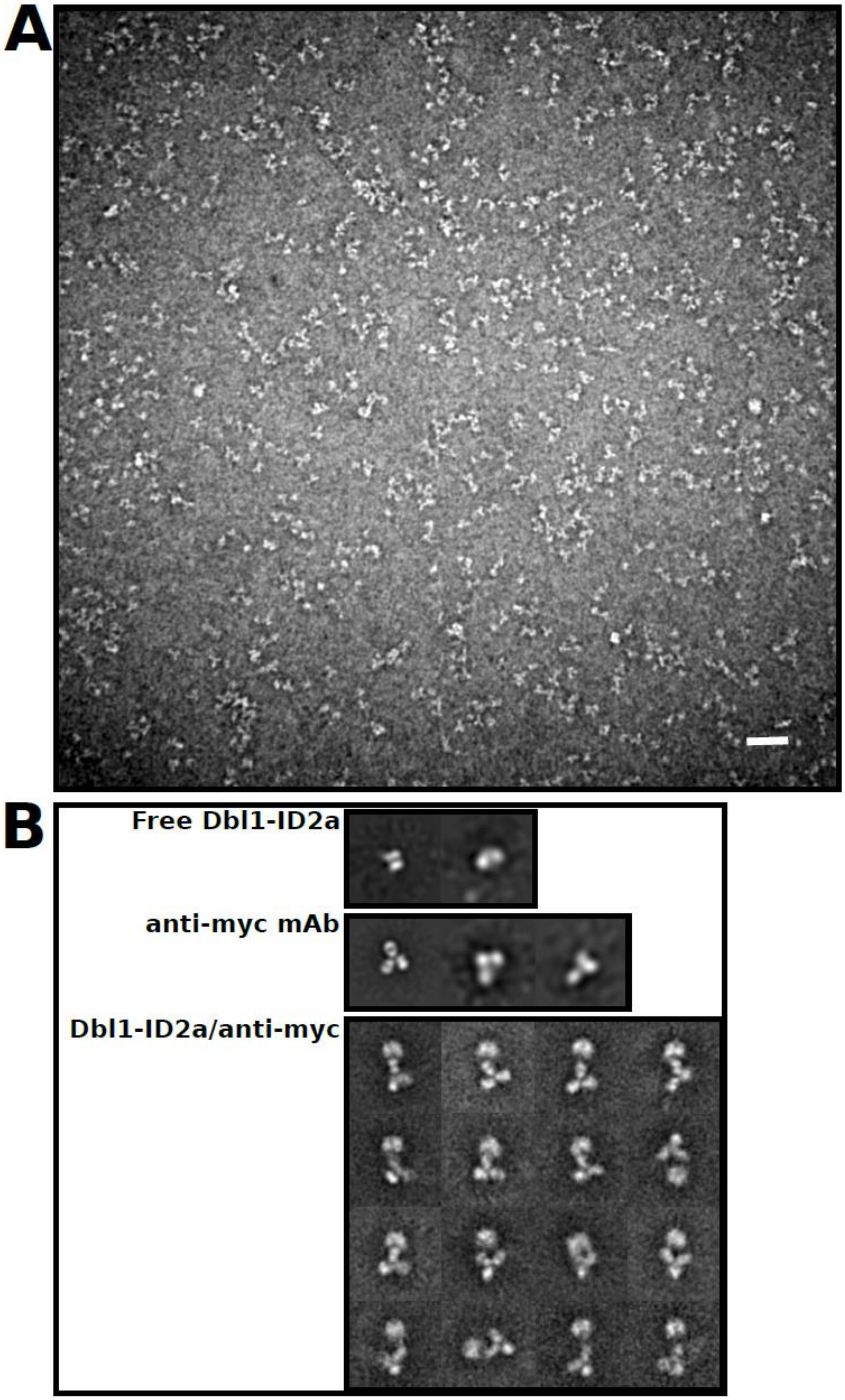
A
B
Free Dbl1-ID2a
anti-myc mAb
Dbl1-ID2a/anti-myc

F**igure S7**. Data and 3-D reconstruction of DBL1x-2IDa:anti cMyc antibody complex. (A) Sample micrograph containing particles of uranyl acetate stained DBL1x-2IDa:anti cMyc antibody. (B) The 2D-classes observed for DBL1x-ID2a alone, anti-cMyc antibody alone and the DBL1x-ID2a:anti-cMyc antibody complex.). The location of the C-terminus could not be unambiguously assigned to any structural feature in this complex. This is likely due to the local conformational freedom of mAb in its interaction with the C-terminal tag of DBL1x-2IDa that are of nearly equal masses and the limited number of views of the complex that arise from preferred orientations in these images.

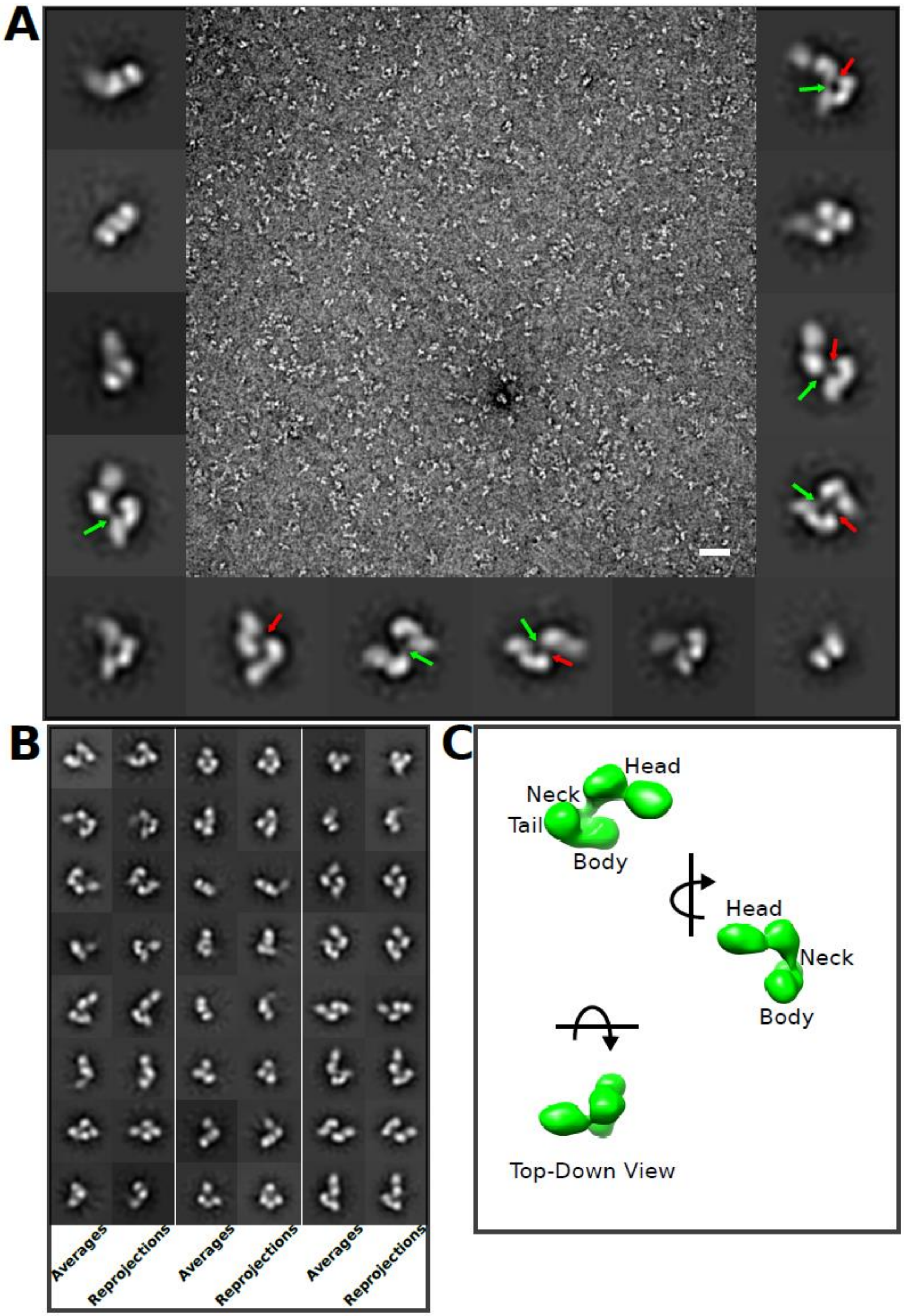
A
B
Averages
Reprojections
Averages
Reprojections
Averages
Reprojections
C
Head
Neck
Tail
Body
Head
Neck
Body
Top-Down View

Figure S8. Data and 3-D reconstruction of DBL3x-DBL6ε. (A) Sample micrograph containing particles of uranyl acetate stained DBL4ε -DBL6ε. A galley of 2D classes surround the micrograph. (B) The top 16 2D-classes paired with the corresponding back projection of the 3D reconstruction. (C) Orthogonal views of the 3D reconstruction.

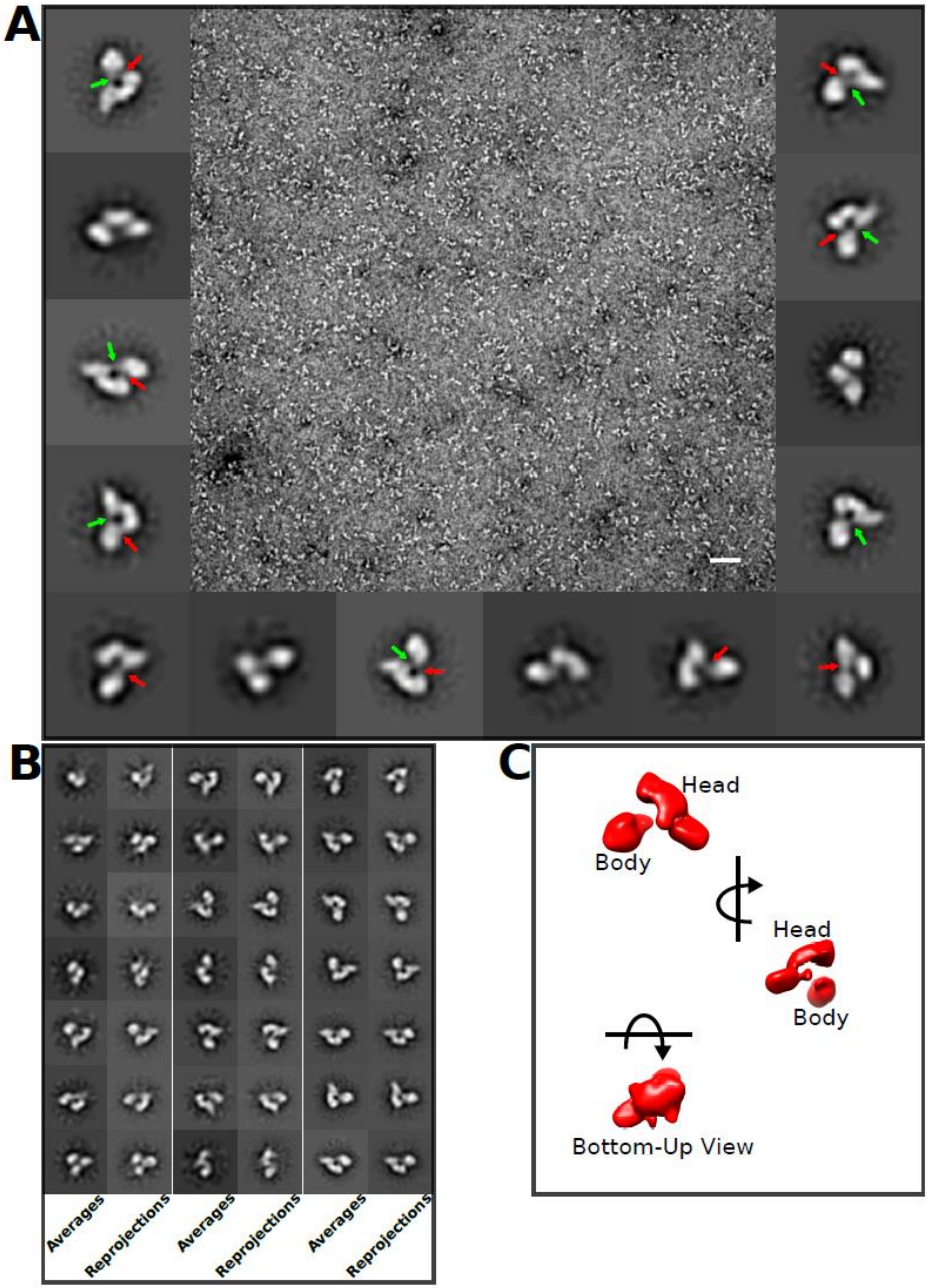
A
B
C
Head
Body
Head
Body
Bottom-Up View
Averages
Reprojections
Averages
Reprojections
Averages
Reprojections

Figure S9. Data and 3-D reconstruction of DBL4ε -DBL6ε. (A) Sample micrograph containing particles of uranyl acetate stained DBL4ε -DBL6ε. A galley of 2D classes surround the micrograph. (B) The top 16 2D-classes paired with the corresponding back projection of the 3D reconstruction. (C) Orthogonal views of the 3D reconstruction.

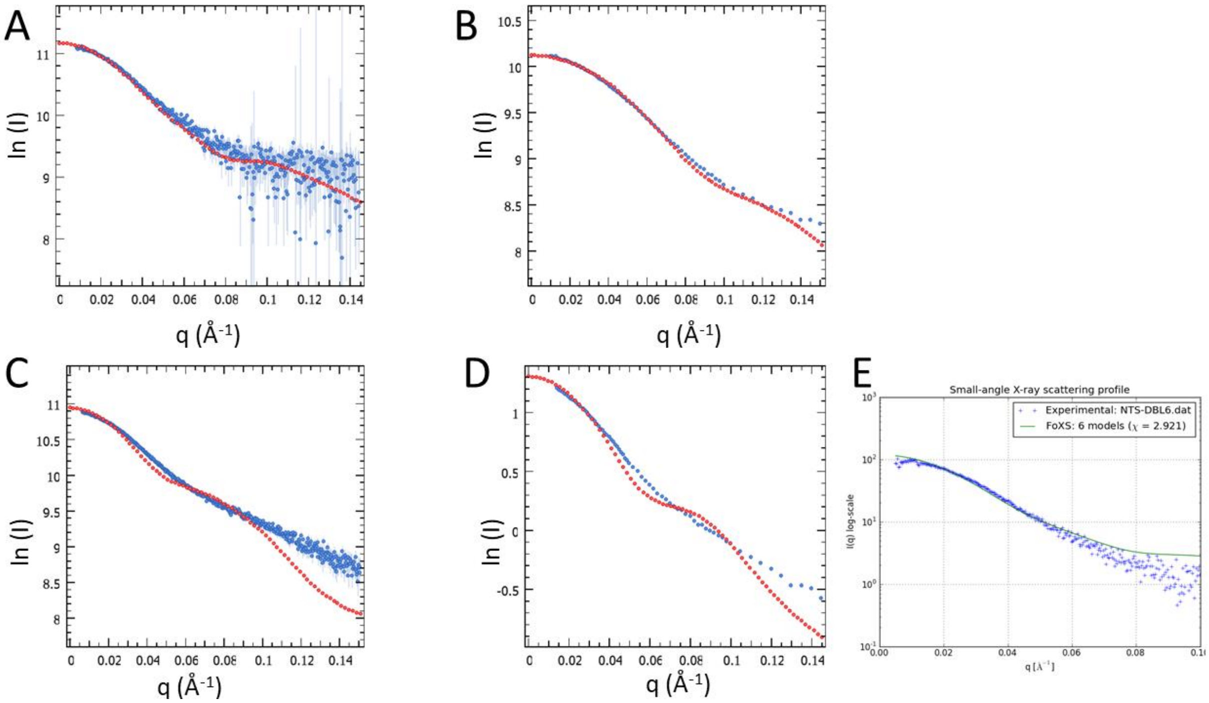

Figure S10 Comparison of EM model based calculated scattering intensities (red) to experimental SAXS intensifies (blue) for the (A) NTS-DBL6ε (B) DBL1x-2IDa (C) DBL3x-DBL6ε (D) DBL4ε-DBL6ε constructs. Error bars corresponding to the SAXS data are drawn as blue vertical lines. (E) Fit of EM based homology model to experimental SAXS curve for NTS-DBL6ε.

NTS-DBL6ε

DKSSIANKIEAYLGAKSDDSKIDQSLKADPSEVQYYGSGGDGYYLRKNICKITVNHGTDSGTNDP
CDRIPPPYGDNDQWKCAIILSKVSEKPENVFVPPRRQRMCINNLEKLNVDKIRDKHAFLADVLLT
ARNEGERIVQNHPDTNSANVCNALERSFADIADIIRGTDLWKGTNSNLEQNLKQMFAKIRENDK
VLQDKYPKDQNYRKLREDWWNANRQKVWEVITCGARSNDLLIKRGWRTSGKSNGDNKLELCR
KCGHYEEKVPTKLDYVPQFLRWLTEWIEDFYREKQNLIDDMERHREECTSEDHKSKEGTSYCST
CKDKCKKYCECVKKWKSEWENQKNKYTELYQQNKNEASQKNTSRYDDYVKDFFKKLEANYA
SLENYIKGDPYFAEYATKLSFILNSADANNPSEKIQKNNDEVCNCNESGIASVEQEQISDPSSNKA
CITHSSIKANKKKVCKHVKLGVRENDKDLRVCVIEHTSLSGVENCCCQDFLRILQENCADNKAG
SSSNGACNNKNQEACEKNLEKVLASLTNCYKCDKCKSEQSKKNNKNWIWKKSSGKEGGLQKE
YANTIGLPPRTQSLCLVVCLDEKGKKTQELKNIRTNSELLKEWIIAAFHEGKNLKPSHEKKNDDN
GKKLCKALEYSFADYGDLIKGTSIWDNEYTKDLELNLQKIFGKLFRKYIKKNNAAEQDTSYSSLD
ELRESWWNTNKKYIWLAMKHGAGMNSTTCCGDGSVTGSGSSCDDIPTIDLIPQYLRFLQEWVE
HFCKQRQEKVKPVIENCKSCKESGGTCNGECKTECKNKCEVYKKFIEDCKGGDGTAGSSWVKR
WDQIYKRYSKYIEDAKRNRKAGTKNCGPSSTTNAAENKCVQSDIDSFFKHLIDIGLTTPSSYLSIV
LDDNICGADKAPWTTYTTYTTTEKCNKETDKSKLQQCNTAVVVNVPSPLGNTPHGYKYACQCK
IPTNEETCDDRKEYMNQWSCGSARTMKRGYKNDNYELCKYNGVDVKPTTVRSNSAKLDDKDV
TFFNLFEQWNKEIQYQIEQYMTNTKISCNNEKNVLSRVSDEAAQPKFSDNERDRNSITHEDKNCK
EKCKCYSLWIEKINDQWDKQKDNYNKFQRKQIYDANKGSQNKKVVSLSNFLFFSCWEEYIQKY
FNGDWSKIKNIGSDTFEFLIKKCGNDSGDGETIFSEKLNNAEKKCKENESTNNKMKSSETSCDCS
EPIYIRGCQPKIYDGKIFPGKGGEKQWICKDTIIHGDTNGACIPPRTQNLCVGELWDKRYGGRSNI
KNDTKESLKQKIKNAIQKETELLYEYHDKGTAIISRNPMKGQKEKEEKNNDSNGLPKGFCHAVQ
RSFIDYKNMILGTSVNIYEYIGKLQEDIKKIIEKGTTKQNGKTVGSGAENVNAWWKGIEGEMWD
AVRCAITKINKKQKKNGTFSIDECGIFPPTGNDEDQSVSWFKEWSEQFCIERLQYEKNIRDACTNN
GQGDKIQGDCKRKCEEYKKYISEKKQEWDKQKTKYENKYVGKSASDLLKENYPECISANFDFIF
NDNIEYKTYYPYGDYSSICSCEQVKYYEYNNAEKKNNKLLCHEKGNDRTWSKKYIKKLENGRT
LEGVYVPPRRQQLCLYELFPIIIKNKNDITNAKKELLETLQIVAEREAYYLWKQYHAHNDATYLA
HKKACCAIRGSFYDLEDIIKGNDLVHDEYTKYIDSKLNEIFDSSNKNDIETKRARTDWWENEAIA
VPNIAGANKADPKTIRQLVWDAMQSGVRKAIDEEKEKKKPNENFPPCMGVQHIGIAKPQFIRWL
EEWTNEFCEKYTKYFEDMKSNCNLRKGADDCDDNSNIECKKACANYANWLNPKRIEWNGMSN
YYNKIYRKSNKESEDGKDYSMIMEPTVIDYLNKRCNGEINGNYICCSCKNIGENSASGTVNKKLQ
KKETQCEDNKGPLDLMNKVLNKMDPKYSEHKMKCTEVYLEHVEEQLKEIDNAIKDYKLYPLDR
CFDDKSKMKVCDLIGDAIGCKHKTKLDELDEWNDVDMRDPYNKYKGVLIPPRRRQLCFSRIVR
GPANLRNLKEFKEEILKGAQSEGKFLGNYYNEDKDKEKALEAMKNSFYDYEYIIKGSDMLTNIQ
FKDIKRKLDRLLEKETNNAEKVDDWWETNKKSIWNAMLCGYKKSGNKIIDPSWCTIPTTETPPQ
FLRWIKEWGTNVCIQKEEHKEYVKSKCSNVANLGAQESESKNCTSEIKKYQEWSRKRSIQWEAI
SEGYKKKYKGMDEFKNTFKNIKEPDANEPNANEYLKKHCSKCPCGFNDMQEITKYTNIGNEAFKQ
IKEQVDIPAELEDVIYRLKHHEYDKGNDYICNKYKNINVNMKKNNDDTWTDLVKNSSDINKGV
LLPPRRKNLFLKIDESDICKYKRDPKLFKDFIYSSAISEVERLKKVYGEAKTKVVHAMKYSFADIG
SIIKGDDMMENNSSDKIGKILGDGVGQNEKRKKWWDMNKYHIWESMLCGYKHAYGNIAENDR
KMLDIPNNDDEHQFLRWFQEWTENFCTKRNELYENMVTACNSAKCNTSNGSVDKKECTEACK
NYANFILIKKKEYQSLNSQYDMNYKETKAEKKESPEYFKDKCNGECSCLSEYFKDETRWKNPYE
TLDDTEVKNNCMCKPPPPASNSGRELEFGPEQKLISEEDLNSAVDHHHHHH

DBL1X-2IDa

DSGTNDPCDRIPPPYGDNDQWKCAIILSKVSEKPENVFVPPRRQRMCINNLEKLNVDKIRDKHAF
LADVLLTARNEGERIVQNHPDTNSANVCNALERSFADIADIIRGTDLWKGTNSNLEQNLKQMFA
KIRENDKVLQDKYPKDQNYRKLREDWWNANRQKVWEVITCGARSNDLLIKRGWRTSGKSNGD

NKLELCRKCGHYEEKVPTKLDYVPQFLRWLTEWIEDFYREKQNLIDDMERHREECTSEDHKSKE
GTSYCSTCKDKCKKYCECVKKWKSEWENQKNKYTELYQQNKNEASQKNTSRYDDYVKDFFK
KLEANYASLENYIKGDPYFAEYATKLSFILNSADANNPSEKIQKNNDEVCNCNESGIASVEQEQIS
DPSSNKACITHSSIKANKKKVCKHVKLGVRENDKDLRVCVIEHTSLSGVENCCCQDFLRILQENC
ADNKAGSSSNGACNNKNQEACEKNLEKVLASLTNCYKCDKCKSEQSKKNNNKNWIWKKSSGKE
GGLQKEYANTIGLPPRTQSLCLVVCLDEKGKKTQELKNIRTNSELLKEWIIAAFHEGKNLKPSHE
KKNDDNGKKLCKALEYSFADYGDLIKGTSIWDNEYTKDLELNLQKIFGKLFRKYIKKNNAAEQD
TSYSSLDELRESWWNTNKKYIWLAMKHGAGMNSTTCCGDGSVTGSGSSCDDIPTIDLIPQYLRF
LQEWVEHFCKQRQEKVKPVIENCKSCKESGGTCNGECKTECKNKCEVYKKFIEDCKGGDGTAG
SSWVKRWDQIYKRYSKYIEDAKRNRKAGTKNCGPSSTTNAAENKCVQSDIDSFFKHLIDIGLTTP
SSYLSIVLDDNICGADKAPWTTYTTYTTTEKCNKETDKSKLQQCNTAVVVNVPSPLGNTPHGYK
YACQCKIPTNEETCDDRKEYMNQWSCGSARTMKRGYKNDNYELCKYNGVDVKPTTVRSNSAK
LDDKDGPEQKLISEEDLNSAVDHHHHHH

ID2b-DBL6ε (used for carbohydrate binding assay)
DDKDVTFFNLFEQWNKEIQYQIEQYMTNTKISCNNEKNVLSRVSDEAAQPKFSDNERDRNSITHE
DKNCKEKCKCYSLWIEKINDQWDKQKDNYNKFQRKQIYDANKGSQNKKVVSLSNFLFFSCWEE
YIQKYFNGDWSKIKNIGSDTFEFLIKKCGNDSGDGETIFSEKLNNAEKKCKENESTNNKMKSSET
SCDCSEPIYIRGCQPKIYDGKIFPGKGGEKQWICKDTIIHGDTNGACIPPRTQNLCVGELWDKRYG
GRSNIKNDTKESLKQKIKNAIQKETELLYEYHDKGTAIISRNPMKGQKEKEEKNNDSNGLPKGFC
HAVQRSFIDYKNMILGTSVNIYEYIGKLQEDIKKIIEKGTTKQNGKTVGSGAENVNAWWKGIEGE
MWDAVRCAITKINKKQKKNGTFSIDECGIFPPTGNDEDQSVSWFKEWSEQFCIERLQYEKNIRDA
CTNNGQGDKIQGDCKRKCEEYKKYISEKKQEWDKQKTKYENKYVGKSASDLLKENYPECISAN
FDFIFNDNIEYKTYYPYGDYSSICSCEQVKYYEYNNAEKKNNKLLCHEKGNDRTWSKKYIKKLE
NGRTLEGVYVPPRRQQLCLYELFPIIIKNKNDITNAKKELLETLQIVAEREAYYLWKQYHAHNDA
TYLAHKKACCAIRGSFYDLEDIIKGNDLVHDEYTKYIDSKLNEIFDSSNKNDIETKRARTDWWEN
EAIAVPNIAGANKADPKTIRQLVWDAMQSGVRKAIDEEKEKKKPNENFPPCMGVQHIGIAKPQFI
RWLEEWTNEFCEKYTKYFEDMKSNCNLRKGADDCDDNSNIECKKACANYANWLNPKRIEWNG
MSNYYNKIYRKSNKESEDGKDYSMIMEPTVIDYLNKRCNGEINGNYICCSCKNIGENSASGTVN
KKLQKKETQCEDNKGPLDLMNKVLNKMDPKYSEHKMKCTEVYLEHVEEQLKEIDNAIKDYKL
YPLDRCFDDKSKMKVCDLIGDAIGCKHKTKLDELDEWNDVDMRDPYNKYKGVLIPPRRRQLCF
SRIVRGPANLRNLKEFKEEILKGAQSEGKFLGNYYNEDKDKEKALEAMKNSFYDYEYIIKGSDM
LTNIQFKDIKRKLDRLLEKETNNAEKVDDWWETNKKSIWNAMLCGYKKSGNKIIDPSWCTIPTT
ETPPQFLRWIKEWGTNVCIQKEEHKEYVKSKCSNVANLGAQESESKNCTSEIKKYQEWSRKRSIQ
WEAISEGYKKYKGMDEFKNTFKNIKEPDANEPNANEYLKKHCSKCPCGFNDMQEITKYTNIGNE
AFKQIKEQVDIPAELEDVIYRLKHHEYDKGNDYICNKYKNINVNMKKNNDDTWTDLVKNSSDIN
KGVLLPPRRKNLFLKIDESDICKYKRDPKLFKDFIYSSAISEVERLKKVYGEAKTKVVHAMKYSF
ADIGSIIKGDDMMENNSSDKIGKILGDGVGQNEKRKKWWDMNKYHIWESMLCGYKHAYGNIA
ENDRKMLDIPNNDDEHQFLRWFQEWTENFCTKRNELYENMVTACNSAKCNTSNGSVDKKECT
EACKNYANFILIKKKEYQSLNSQYDMNYKETKAEKKESPEYFKDKCNGECSCLSEYFKDETRWK
NPYETLDDTEVKNNCMCKPPPPASNSGRELEFGPEQKLISEEDLNSAVDHHHHHH

ID2b-DBL6ε (used for all studies except carbohydrate binding assays)
METDTLLLWVLLLWVPGSTGDGTDDKDVTFFNLFEQWNKEIQYQIEQYMTNTKISCNNEKNVL
SRVSDEAAQPKFSDNERDRNSITHEDKNCKEKCKCYSLWIEKINDQWDKQKDNYNKFQRKQIY
DANKGSQNKKVVSLSNFLFFSCWEEYIQKYFNGDWSKIKNIGSDTFEFLIKKCGNDSGDGETIFSE
KLNNAEKKCKENESTNNKMKSSETSCDCSEPIYIRGCQPKIYDGKIFPGKGGEKQWICKDTIIHGD
TNGACIPPRTQNLCVGELWDKRYGGRSNIKNDTKESLKQKIKNAIQKETELLYEYHDKGTAIISR
NPMKGQKEKEEKNNDSNGLPKGFCHAVQRSFIDYKNMILGTSVNIYEYIGKLQEDIKKIIEKGTT
KQNGKTVGSGAENVNAWWKGIEGEMWDAVRCAITKINKKQKKNGTFSIDECGIFPPTGNDEDQ
SVSWFKEWSEQFCIERLQYEKNIRDACTNNGQGDKIQGDCKRKCEEYKKYISEKKQEWDKQKT

KYENKYVGKSASDLLKENYPECISANFDFIFNDNIEYKTYYPYGDYSSICSCEQVKYYEYNNAEK
KNNKLLCHEKGNDRTWSKKYIKKLENGRTLEGVYVPPRRQQLCLYELFPIIIKNKNDITNAKKEL
LETLQIVAEREAYYLWKQYHAHNDATYLAHKKACCAIRGSFYDLEDIIKGNDLVHDEYTKYIDS
KLNEIFDSSNKNDIETKRARTDWWENEAIAVPNIAGANKADPKTIRQLVWDAMQSGVRKAIDEE
KEKKKPNENFPPCMGVQHIGIAKPQFIRWLEEWTNEFCEKYTKYFEDMKSNCNLRKGADDCDD
NSNIECKKACANYANWLNPKRIEWNGMSNYYNKIYRKSNKESEDGKDYSMIMEPTVIDYLNKR
CNGEINGNYICCSCKNIGENSASGTVNKKLQKKETQCEDNKGPLDLMNKVLNKMDPKYSEHKM
KCTEVYLEHVEEQLKEIDNAIKDYKLYPLDRCFDDKSKMKVCDLIGDAIGCKHKTKLDELDEWN
DVDMRDPYNKYKGVLIPPRRRQLCFSRIVRGPANLRNLKEFKEEILKGAQSEGKFLGNYYNEDK
DKEKALEAMKNSFYDYEYIIKGSDMLTNIQFKDIKRKLDRLLEKETNNAEKVDDWWETNKKSI
WNAMLCGYKKSGNKIIDPSWCTIPTTETPPQFLRWIKEWGTNVCIQKEEHKEYVKSKCSNVANL
GAQESESKNCTSEIKKYQEWSRKRSIQWEAISEGYKKYKGMDEFKNTFKNIKEPDANEPNANEY
LKKHCSKCPCGFNDMQEITKYTNIGNEAFKQIKEQVDIPAELEDVIYRLKHHEYDKGNDYICNKY
KNINVNMKKNNDDTWTDLVKNSSDINKGVLLPPRRKNLFLKIDESDICKYKRDPKLFKDFIYSSA
ISEVERLKKVYGEAKTKVVHAMKYSFADIGSIIKGDDMMENNSSDKIGKILGDGVGQNEKRKK
WWDMNKYHIWESMLCGYKHAYGNIAENDRKMLDIPNNDDEHQFLRWFQEWTENFCTKRNEL
YENMVTACNSAKCNTSNGSVDKKECTEACKNYANFILIKKKEYQSLNSQYDMNYKETKAEKKE
SPEYFKDKCNGECSCLSEYFKDETRWKNPYETLDDTEVKNNCMCKPPPPASNSGRELEFGPENL
YFQGNSAVDDYKDHDGDYKDHDIDYKDDDDKLEVLFQGTGIHHHHHH

DBL3X-DBL6ε
DGTMKSSETSCDCSEPIYIRGCQPKIYDGKIFPGKGGEKQWICKDTIIHGDTNGACIPPRTQNLCV
GELWDKRYGGRSNIKNDTKESLKQKIKNAIQKETELLYEYHDKGTAIISRNPMKGQKEKEEKNN
DSNGLPKGFCHAVQRSFIDYKNMILGTSVNIYEYIGKLQEDIKKIIEKGTTKQNGKTVGSGAENV
NAWWKGIEGEMWDAVRCAITKINKKQKKNGTFSIDECGIFPPTGNDEDQSVSWFKEWSEQFCIE
RLQYEKNIRDACTNNGQGDKIQGDCKRKCEEYKKYISEKKQEWDKQKTKYENKYVGKSASDLL
KENYPECISANFDFIFNDNIEYKTYYPYGDYSSICSCEQVKYYEYNNAEKKNNKLLCHEKGNDRT
WSKKYIKKLENGRTLEGVYVPPRRQQLCLYELFPIIIKNKNDITNAKKELLETLQIVAEREAYYL
WKQYHAHNDATYLAHKKACCAIRGSFYDLEDIIKGNDLVHDEYTKYIDSKLNEIFDSSNKNDIET
KRARTDWWENEAIAVPNIAGANKADPKTIRQLVWDAMQSGVRKAIDEEKEKKKPNENFPPCMG
VQHIGIAKPQFIRWLEEWTNEFCEKYTKYFEDMKSNCNLRKGADDCDDNSNIECKKACANYAN
WLNPKRIEWNGMSNYYNKIYRKSNKESEDGKDYSMIMEPTVIDYLNKRCNGEINGNYICCSCKN
IGENSASGTVNKKLQKKETQCEDNKGPLDLMNKVLNKMDPKYSEHKMKCTEVYLEHVEEQLK
EIDNAIKDYKLYPLDRCFDDKSKMKVCDLIGDAIGCKHKTKLDELDEWNDVDMRDPYNKYKG
VLIPPRRRQLCFSRIVRGPANLRNLKEFKEEILKGAQSEGKFLGNYYNEDKDKEKALEAMKNSFY
DYEYIIKGSDMLTNIQFKDIKRKLDRLLEKETNNAEKVDDWWETNKKSIWNAMLCGYKKSGNK
IIDPSWCTIPTTETPPQFLRWIKEWGTNVCIQKEEHKEYVKSKCSNVANLGAQESESKNCTSEIKK
YQEWSRKRSIQWEAISEGYKKYKGMDEFKNTFKNIKEPDANEPNANEYLKKHCSKCPCGFNDM
QEITKYTNIGNEAFKQIKEQVDIPAELEDVIYRLKHHEYDKGNDYICNKYKNINVNMKKNNDDT
WTDLVKNSSDINKGVLLPPRRKNLFLKIDESDICKYKRDPKLFKDFIYSSAISEVERLKKVYGEAK
TKVVHAMKYSFADIGSIIKGDDMMENNSSDKIGKILGDGVGQNEKRKKWWDMNKYHIWESML
CGYKHAYGNIAENDRKMLDIPNNDDEHQFLRWFQEWTENFCTKRNELYENMVTACNSAKCNT
SNGSVDKKECTEACKNYANFILIKKKEYQSLNSQYDMNYKETKAEKKESPEYFKDKCNGECSCL
SEYFKDETRWKNPYETLDDTEVKNNGPENLYFQGNSAVDDYKDHDGDYKDHDIDYKDDDDKL
EVLFQGISVPSIPPDVSGFSIGIHHHHHH

DBL4ε-DBL6ε (for carbohydrate binding assays)
DQVKYYEYNNAEKKNNKLLCHEKGNDRTWSKKYIKKLENGRTLEGVYVPPRRQQLCLYELFPII
IKNKNDITNAKKELLETLQIVAEREAYYLWKQYHAHNDATYLAHKKACCAIRGSFYDLEDIIKG
NDLVHDEYTKYIDSKLNEIFDSSNKNDIETKRARTDWWENEAIAVPNIAGANKADPKTIRQLVW
DAMQSGVRKAIDEEKEKKKPNENFPPCMGVQHIGIAKPQFIRWLEEWTNEFCEKYTKYFEDMKS

NCNLRKGADDCDDNSNIECKKACANYANWLNPKRIEWNGMSNYYNKIYRKSNKESEDGKDYS
MIMEPTVIDYLNKRCNGEINGNYICCSCKNIGENSASGTVNKKLQKKETQCEDNKGPLDLMNKV
LNKMDPKYSEHKMKCTEVYLEHVEEQLKEIDNAIKDYKLYPLDRCFDDKSKMKVCDLIGDAIG
CKHKTKLDELDEWNDVDMRDPYNKYKGVLIPPRRRQLCFSRIVRGPANLRNLKEFKEEILKGAQ
SEGKFLGNYYNEDKDKEKALEAMKNSFYDYEYIIKGSDMLTNIQFKDIKRKLDRLLEKETNNAE
KVDDWWETNKKSIWNAMLCGYKKSGNKIIDPSWCTIPTTETPPQFLRWIKEWGTNVCIQKEEHK
EYVKSKCSNVANLGAQESESKNCTSEIKKYQEWSRKRSIQWEAISEGYKKYKGMDEFKNTFKNI
KEPDANEPNANEYLKKHCSKCPCGFNDMQEITKYTNIGNEAFKQIKEQVDIPAELEDVIYRLKHH
EYDKGNDYICNKYKNINVNMKKNNDDTWTDLVKNSSDINKGVLLPPRRKNLFLKIDESDICKYK
RDPKLFKDFIYSSAISEVERLKKVYGEAKTKVVHAMKYSFADIGSIIKGDDMMENNSSDKIGKIL
GDGVGQNEKRKKWWDMNKYHIWESMLCGYKHAYGNIAENDRKMLDIPNNDDEHQFLRWFQE
WTENFCTKRNELYENMVTACNSAKCNTSNGSVDKKECTEACKNYANFILIKKKEYQSLNSQYD
MNYKETKAEKKESPEYFKDKCNGECSCLSEYFKDETRWKNPYETLDDTEVKNNGPEQKLISEED
LNSAVDHHHHHH

DBL4ε-DBL6ε (for carbohydrate binding assays)
DQVKYYEYNNAEKKNNKLLCHEKGNDRTWSKKYIKKLENGRTLEGVYVPPRRQQLCLYELFPII
IKNKNDITNAKKELLETLQIVAEREAYYLWKQYHAHNDATYLAHKKACCAIRGSFYDLEDIIKG
NDLVHDEYTKYIDSKLNEIFDSSNKNDIETKRARTDWWENEAIAVPNIAGANKADPKTIRQLVW
DAMQSGVRKAIDEEKEKKKPNENFPPCMGVQHIGIAKPQFIRWLEEWTNEFCEKYTKYFEDMKS
NCNLRKGADDCDDNSNIECKKACANYANWLNPKRIEWNGMSNYYNKIYRKSNKESEDGKDYS
MIMEPTVIDYLNKRCNGEINGNYICCSCKNIGENSASGTVNKKLQKKETQCEDNKGPLDLMNKV
LNKMDPKYSEHKMKCTEVYLEHVEEQLKEIDNAIKDYKLYPLDRCFDDKSKMKVCDLIGDAIG
CKHKTKLDELDEWNDVDMRDPYNKYKGVLIPPRRRQLCFSRIVRGPANLRNLKEFKEEILKGAQ
SEGKFLGNYYNEDKDKEKALEAMKNSFYDYEYIIKGSDMLTNIQFKDIKRKLDRLLEKETNNAE
KVDDWWETNKKSIWNAMLCGYKKSGNKIIDPSWCTIPTTETPPQFLRWIKEWGTNVCIQKEEHK
EYVKSKCSNVANLGAQESESKNCTSEIKKYQEWSRKRSIQWEAISEGYKKYKGMDEFKNTFKNI
KEPDANEPNANEYLKKHCSKCPCGFNDMQEITKYTNIGNEAFKQIKEQVDIPAELEDVIYRLKHH
EYDKGNDYICNKYKNINVNMKKNNDDTWTDLVKNSSDINKGVLLPPRRKNLFLKIDESDICKYK
RDPKLFKDFIYSSAISEVERLKKVYGEAKTKVVHAMKYSFADIGSIIKGDDMMENNSSDKIGKIL
GDGVGQNEKRKKWWDMNKYHIWESMLCGYKHAYGNIAENDRKMLDIPNNDDEHQFLRWFQE
WTENFCTKRNELYENMVTACNSAKCNTSNGSVDKKECTEACKNYANFILIKKKEYQSLNSQYD
MNYKETKAEKKESPEYFKDKCNGECSCLSEYFKDETRWKNPYETLDDTEVKNNGPENLYFQGN
SAVDDYKDHDGDYKDHDIDYKDDDDKLEVLFQGTGIHHHHHH

Table S1. Amino acid sequences of constructs described in the text as defined in Figure 1A. The regions underlined show the differences corresponding to the affinity tag for similar constructs

DBL1x-2IDa (flag)	GGGCCCGAGAACCTGTAC	GTCCTTGTCGTCCAGCTTAG
DBL1x-2IDb (flag)	GGGCCCGAGAACCTGTAC	CTTGTTGTTGGTGGACTCGTTTT
ID2b-DBL6ε (flag)	GACGACAAGGACGTCACC	GGTACCGTCACCAGTGGA
DBL3x-DBL6ε flag	ATGAAGTCCTCTGAAACCTCCT	GTCACCAGTGGAACCTGG
DBL4ε-DBL6ε myc	CAAGTGAAGTACTACGAGTAC	GTCACCAGTGGAACCTGG
Removal of N-terminal cloning artefact from vector	GTC ACC AGT GGA ACC TGG AAC CGGT	GGT ACC GAA TTC GGG CCC
Truncation of C-Terminus	GAACAAAAACTCATCTCAGAAG	GTTGTTCTTGACTTCGGTG
Removal of ApaI site and cmyc tag-introduction of EcoRV site	TCCATCATCATCATCATCATTGAG TTTAAACC	TATCGGTACCGTCACCAGTG

GBLOCK Amino acids 1-58 + KpnI	AAGTCCTCTATCGCCAACAAGATCGAAGCCTACCTGGGAGC CAAGTCTGACGACTCTAAGATCGACCAGTCTCTGAAGGCCG ACCCCTCTGAAGTCCAGTACTACGGATCTGGTGGCGACGGT TACTACCTGAGGAAGAACATCTGCAAGATCACCGTCAACCA CGGTACCGACTCTGGG
GBLOCK EcoRI+ApaI site + TEV protease + 3XFLAG	GAATTCGGGCCCGAGAACCTGTACTTCCAAGGCAATAGCGC CGTCGACGACTACAAAGACCATGACGGTGATTATAAAGATC ATGACATCGATTACAAGGATGACGATGACAAGCTGGAAGTT CTGTTCCAGGGGACCGGG

Table S2 Primers used to generate constructs.

	NTS-DBL6ε	DBL3x-DBL6ε	DBL4ε-DBL6ε	DBL1x-ID2a
Experimental Date	11-01-2017	11-01-2017	07-08-2018	03-05-2018
Location	APS	APS	NSLSII	NSLSII
Beamline/instrument	18-ID	18-ID	16-ID	16-ID
instrument	Pilatus 1M	Pilatus 1M	Pilatus 1M (SAXS) and 3K (WAXS)	Pilatus 1M (SAXS) and 3K (WAXS)
SEC flow rate (mls/min)	0.5	0.5	0.5	0.5
Cell temperature	RT	RT	RT	RT
Protein concentration (mg/ml)			10.2	7.5
Number of residues, including tags	2671	1483	1095	1016
Molecular weight (calculated)	309845	173423	128802	116489

Table S3 Experimental details for SAXS data collection

	Residue number	Template	Matched residues	% Identity (similarity)	Residues modeled	zDOPE*
DBL1x	10-388	2YK0	31-434	30 (54)	10-410	-0.42 (-0.30)
ID1	372-580	NT				
DBL2x	581-906	2XU0_A	132-447	23 (49)	569-905	0.04 (0.08)
ID2a	907-1078	NT				
ID2b	1079-1149	4P1T	1833-1905	22 (52)		
	1150-1216	NT				
DBL3x-DBL4ε	1207-1944	4P1T	1215-1949	83 (89)	1205-1944	-0.55 (-0.55)
	1965-1978	NT				
DBL5ε	1979-2290	3VUV_A	41-446	31 (54)	1979-2283	-0.55 (-0.51)
	2291-2332	NT				
DBL6ε	2333-2364	2WAU_A	2333-2634	99 (99)	2350-2634	-1.89 (-1.88)

Table S4. Regions modelled using Modeller and templates used.

NT = no template; GA341 = 1 in all cases
